# How pollen tubes fight for food: the impact of sucrose carriers and invertases of *Arabidopsis thaliana* on pollen development and pollen tube growth

**DOI:** 10.3389/fpls.2023.1063765

**Published:** 2023-06-22

**Authors:** Jessica Seitz, Theresa Maria Reimann, Carolin Fritz, Carola Schröder, Johanna Knab, Walter Weber, Ruth Stadler

**Affiliations:** ^1^ Molecular Plant Physiology, Department of Biology, Friedrich-Alexander University Erlangen-Nuremberg, Erlangen, Germany; ^2^ Cell Biology, Department of Biology, Friedrich-Alexander University Erlangen-Nuremberg, Erlangen, Germany

**Keywords:** *Arabidopsis thaliana*, AtSUC1, AtVI2, carbohydrate storage, esculin, pollen tubes, sucrose transporters, vacuolar invertases

## Abstract

Pollen tubes of higher plants grow very rapidly until they reach the ovules to fertilize the female gametes. This growth process is energy demanding, however, the nutrition strategies of pollen are largely unexplored. Here, we studied the function of sucrose transporters and invertases during pollen germination and pollen tube growth. RT-PCR analyses, reporter lines and knockout mutants were used to study gene expression and protein function in pollen. The genome of Arabidopsis thaliana contains eight genes that encode functional sucrose/H^+^ symporters. Apart from *AtSUC2*, which is companion cell specific, all other *AtSUC* genes are expressed in pollen tubes. AtSUC1 is present in developing pollen and seems to be the most important sucrose transporter during the fertilization process. Pollen of an *Atsuc1 knockout* plant contain less sucrose and have defects in pollen germination and pollen tube growth. The loss of other sucrose carriers affects neither pollen germination nor pollen tube growth. A multiple knockout line *Atsuc1Atsuc3Atsuc8Atsuc9* shows a phenotype that is comparable to the Atsuc1 mutant line. Loss of AtSUC1 can`t be complemented by AtSUC9, suggesting a special function of AtSUC1. Besides sucrose carriers, pollen tubes also synthesize monosaccharide carriers of the AtSTP family as well as invertases. We could show that *AtcwINV2* and *AtcwINV4* are expressed in pollen, *AtcwINV1* in the transmitting tissue and *AtcwINV5* in the funiculi of the ovary. The vacuolar invertase *AtVI2* is also expressed in pollen, and a knockout of *AtVI2* leads to a severe reduction in pollen germination. Our data indicate that AtSUC1 mediated sucrose accumulation during late stages of pollen development and cleavage of vacuolar sucrose into monosaccharides is important for the process of pollen germination.

## Introduction

The pollen represents the male gametophyte of seed plants. When a pollen grain lands on an appropriate stigma, it hydrates and produces a pollen tube that grows through the stigma, the style, and the ovary until it reaches the ovules. This tip-growth process is very fast and requires the pollen to efficiently synthesize proteins, cell membrane, and cell wall material (Dresselhaus & Franklin-Tong, 2013). The components are transported through an extensive vesicular network to the growing tip (Guan et al., 2013; Hao et al., 2022). Interestingly, it is not yet well understood which substances pollen use as an energy source for this demanding growth process.

Generally, pollen may be pre-loaded with carbohydrates (Speranza et al., 1997) as well as lipids during their development (Regan & Moffatt, 1990). *Arabidopsis thaliana* pollen contain lipid droplets, which could be used not only for membrane biosynthesis but also as an energy source (Bai et al., 2023). Another strategy for pollen nutrition might be the import of sugars during pollen tube growth. This strategy assumes the presence of nutrients, most likely sucrose, in the apoplasmic fluid of the pistil. Sucrose is mainly produced in leaves and delivered to the flowers *via* the phloem, where it reaches concentrations of up to 0.3 M (Deeken et al., 2002). Studies with phloem mobile GFP synthesized in companion cells demonstrated that phloem unloading in *Arabidopsis thaliana* pistils follows an entirely symplasmic pathway. As GFP accumulated in all cells of the pistil except the fully developed ovules (Werner et al., 2011), these studies indicate that the cells along the pathway of growing pollen tubes may contain high amounts of sucrose. Sucrose export from the cytosol of these cells into the apoplasm might then be mediated by transporters of the SUGAR WILL EVENTUALLY BE EXPORTED (SWEET) family (Chen et al., 2012). The sucrose carrier AtSWEET9 is present in papillae cells of the style and AtSWEET10 was detected in the transmitting tissue of style and ovary (Lin et al., 2014; Rottmann et al., 2018b). Their presence in these cells could lead to a high sucrose concentration in the cell wall area of the stigma and the transmitting tissue, although there are no solid data available on actual sugar concentrations in the apoplasm of the pistil so far. Pollen are not connected to other cells *via* plasmodesmata. Sugar uptake into pollen my occur *via* endocytosis or *via* active sugar/H^+^ cotransporters (Sauer et al., 1994; Etxeberria et al., 2005). Sucrose can be taken up either as disaccharide or after cleavage by apoplasmic invertases as fructose and glucose. Six *SUGAR TRANSPORTER PROTEIN* (*STP*) genes, which encode monosaccharide/H^+^ cotransporters, are expressed in pollen tubes (Rottmann et al., 2018b). Interestingly, the knockout of all six *AtSTP* genes in a sextuple knockout line led to enhanced pollen tube growth on medium containing glucose, however, the knockout plants were fully fertile and produced a normal amount of seeds. This observation led to the hypothesis that glucose is rather a signal that regulates pollen tube growth than a nutrient that promotes growth (Rottmann et al., 2018b).

The presence of sucrose carriers in pollen tubes is less well studied. The genome of *Arabidopsis thaliana* includes nine *SUCROSE TRANSPORTER* genes (*AtSUC1-9*). *AtSUC7* is a pseudogene, all other *AtSUC* genes code for active sucrose/H^+^ cotransporters. They catalyze the transport of sucrose or maltose with *K*_M_ values between 0.2 – 0.8 mM for sucrose (Sauer & Stolz, 1994; Ludwig et al., 2000; Meyer et al., 2000; Weise et al., 2000; Sauer et al., 2004; Rottmann et al., 2018a). AtSUC4 is localized in the tonoplast and transports sucrose from the vacuole into the cytosol (Endler et al., 2006; Schulz et al., 2011; Schneider et al., 2012a). All other AtSUCs reside in the plasma membrane. Several different groups used reporter gene approaches to study the expression of *AtSUC* genes and detected a promoter activity in pollen tubes (Stadler et al., 1999; Schulze et al., 2000; Meyer et al., 2004; Sivitz et al., 2008; Rottmann et al., 2018a), indicating that sucrose transporters are important for pollen tube growth. However, several of the reporter gene lines in the papers mentioned above miss the genomic sequence and the introns of the gene of interest. Reporter often don`t show the *in vivo* situation of expression if the introns are missing (Fiume et al., 2004; Rose, 2004; Sivitz et al., 2007; Weise et al., 2008; Rottmann et al., 2018a). Therefore, we decided to investigate the presence of SUCs in pollen tubes using reporter lines that include the genomic sequence of the genes of interest.

Sucrose can be cleaved by invertases or by sucrose synthases (Stein & Granot, 2019). Invertases can be divided into cell wall invertases (cwINV), alkaline/neutral invertases (A/N-INV), and vacuolar invertases (VI) based on their biochemical properties (solubility, pH optima, isoelectric point) and their subcellular localization (Sturm, 1999; Koch, 2004; Roitsch & González, 2004; Ruan et al., 2010; Ruan, 2014). Phylogenetically, cell wall invertases are closely related to vacuolar invertases, but distant from alkaline/neutral invertases (Ruan et al., 2010). Cell wall and vacuolar invertases also share some biochemical properties. They cleave sucrose in glucose and fructose and are most efficient in acidic environments with a pH between 4.5 and 5.0. In *Arabidopsis thaliana* six genes encode cell wall invertases, *AtcwINV1* - *AtcwINV6*. However, the enzymes AtcwINV3 and AtcwINV6 are catalytically inactive invertases (Sherson et al., 2003; De Coninck et al., 2005). Cell wall invertases are expressed in source tissues, for example in leaves, but also in sink tissues including pollen (Tymowska-Lalanne & Kreis, 1998; Sherson et al., 2003; Hirsche et al., 2009; Ruhlmann et al., 2010; Wang & Ruan, 2012; Liao et al., 2020; Li et al., 2021). There are two genes that code for vacuolar invertases in *Arabidopsis thaliana*: *AtVI1* and *AtVI2* and both vacuolar invertases play a role in plant and seed development (Vu et al., 2020).

In the present paper we analyzed the role of sucrose transporters as well as invertases for the fertility of pollen of *Arabidopsis thaliana*. We used reporter gene lines for invertases and for the sucrose carriers AtSUC1, 3, 4, 5, 6, 8 and 9 containing not only promoter sequences but also genomic sequences together with the introns of the respective genes. We didn`t investigate *AtSUC7* because it is a pseudogene and *AtSUC2* because it is known to be companion cell specific. The *AtSUC2* promotor is well established in the scientific community as a tool to drive companion cell specific expression or as a marker for analyses of companion cell transcriptomes (summarized in Stadler & Sauer, 2019). Our data indicate a dominant role of the sucrose transporter AtSUC1 and the vacuolar invertase AtVI2 during reproduction. Loss of AtSUC1 led to a reduced sucrose storage in mature pollen, which couldn’t be complemented by expression of *AtSUC9* under the control of the *AtSUC1* promoter. Loss of the vacuolar invertase AtVI2 caused a reduced pollen germination, indicating that cleavage of vacuolar sucrose by AtVI2 promotes pollen germination.

## Materials and methods

### Strains, growth conditions, and genotyping

All plant lines used in this study are listed in **Supplementary Table 1**. *Arabidopsis thaliana* (*Arabidopsis thaliana* ecotype Columbia-0 (Col-0)) was grown in the greenhouse in potting soil or under long-day conditions (16 h of light/8 h of dark) at 22°C and 60% humidity in the phytochamber. The T-DNA insertion lines *Atsuc1* (SM3_19978; John-Innes-Centre), *Atsuc3* (GABIKat_325D02; Max-Planck-Institute), *Atsuc8* (SALK_066671; Salk Institute), *Atsuc9* (SALK_050102; Salk Institute), *Atvi2-1* (SALK_011312; Alonso et al., 2003), *Atvi2-2* (SALK_100813; Alonso et al., 2003), *Atcwinv2-1* (SALK_068113; Alonso et al., 2003), *Atcwinv4-2* (SALK_130163; Alonso et al., 2003) and *Atcwinv5* (SALK_GK849H10; Li et al., 2003) were obtained from the Nottingham Stock Centre (https://Arabidopsisthaliana.info/). The single knockout lines *Atsuc4.1* (WiscDsLox450E10; Schneider et al., 2012a) and *Atsuc5.4* (SALK_367_D07; Pommerrenig et al., 2013) were kindly provided by Norbert Sauer (Molecular Plant Physiology, Friedrich-Alexander University of Erlangen-Nuremberg). Homozygous plants were identified by genotyping with the primers listed in **Supplementary Table 2**. PCR products obtained with the respective primer pairs for the mutant allele (**Supplementary Table 2**) were sequenced for the determination of the positions of the T-DNA insertions. The homozygous *Atsuc1Atsuc3Atsuc8Atsuc9* quadruple and the *Atcwinv2-1Atcwinv4-2Atcwinv5* triple knockout lines were generated by crosspollination of the respective knockout lines and PCR-based genotyping of the F2 descendants. The *Attmt1Attmt2* double knockout line (Wormit et al., 2006) was kindly provided by Ekkehart Neuhaus (Division of Plant Physiology, University of Kaiserslautern). *Arabidopsis thaliana* plants were transformed *via* the floral dip method with the *Agrobacterium tumefaciens* strain GV3101 (Holsters et al., 1980; Clough and Bent, 1998). For all cloning steps the *Escherichia coli* strain DH5α (Hanahan, 1983) was used.

### RNA isolation, reverse transcription, RT-PCR, and qPCR

For mRNA isolation, pollen, virgin stigmata and stigmata with semi-*in vivo* grown pollen tubes were placed on pollen germination medium and germinated for at least 5 h. After pollen germination, pollen and/or stigmata of about 30 flowers were rinsed from the cellulose membrane in an Eppendorf tube with 100 µl extraction buffer of the PicoPure™ RNA Isolation Kit (Arcturus) and frozen in liquid nitrogen. After thawing, the samples were incubated for 30 min at 42°C, centrifuged (2 min, 3.000 g, RT) and the supernatant transferred to a new Eppendorf tube. An equal volume of 70% ethanol was added to the supernatant. The further steps correspond to the method listed in the instructions of the kit. Finally, the mRNA was eluted with 11-13 μl of elution buffer and immediately afterwards transcribed into cDNA. For reverse transcription reactions, the QuantiTect^®^ Reverse Transcription Kit (Qiagen) was used. For detection of *AtSUC1 – AtSUC9*, *AtcwINV2*, *AtcwINV4*, *AtcwINV5*, and *AtVI2* transcripts with wild type (WT) or mutant cDNA as template the specific primers in **Supplementary Tables 3, 4** were used for PCR reactions. An *ACTIN2*-specific PCR was performed as a positive control. For the amplification of amplicons 39 cycles were used. RT-PCR analyses with total mRNA of homozygous flowers or pollen tubes confirmed the loss of full-length transcripts of the respective T-DNA insertion lines. In case of *Atsuc8* transcripts of *AtSUC8* could be amplified after the T-DNA insertion but resulting in a functional protein is very unlikely because of frame shift and silencing effect from the T-DNA insertion (Daxinger et al., 2008). For the analyses of p*AtSUC1:AtSUC1*g, p*AtSUC9:AtSUC9*g, and p*AtSUC1:AtSUC9*g expression in pollen tubes grown *in vitro* of *Atsuc1* and *AtSUC1* complementation lines gene specific primers were used (**Supplementary Table 5**). Relative expression of *AtSUC1*, *AtSUC3*, *AtSUC5*, *AtSUC6*, *AtSUC8* and *AtSUC9* in pollen tubes was measured *via* qRT-PCR using the SYBR^®^ Green Kit (Agilent, Santa Clara, CA, USA) and the Rotor-Gene Q Cycler (Qiagen). Primers in **Supplementary Table 6** were used for the detection of the respective transcripts. Relative *AtSUC*s expression levels were normalized by comparison with *UBI10* which was amplified with primer pair UBQ10 + 1066fw and UBQ10 + 1130rev (**Supplementary Table 6**) and calculated according to the 2–ΔΔCT (Livak) method.

### Cloning of reporter gene constructs

All plasmid constructs used in this study are listed in **Supplementary Table 7**. The p*AtSUC1*:*AtSUC1*g-*GUS* reporter plants (Sivitz et al., 2008) were kindly provided by John M. Ward (Department of Plant Biology, University of Minnesota). p*AtSUC2*:*GUS* reporter plants (Schneidereit et al., 2008) were kindly provided by Norbert Sauer (Molecular Plant Physiology, Friedrich-Alexander University of Erlangen-Nuremberg). For generating the p*AtSUC3*:At*SUC3*g reporter gene lines a 2174 bp promoter fragment and one part of the genomic sequence was amplified with the primer pair AtSUC3-2174fw+CACC and AtSUC3g+1277rev (**Supplementary Table 8**), yielding a 3451 bp insert and cloned into *pENTR/D-TOPO* (Invitrogen). The remaining genomic sequence including the introns was amplified with the primer pair AtSUC3g+1199fw and AtSUC3g+3748rev+A+AscI (**Supplementary Table 8**). This resulting 2549 bp fragment was inserted downstream of the first part of *AtSUC3 via* an internal *Pst*I and the attached *Asc*I site. The complete p*AtSUC3*:*AtSUC3*g construct was inserted by LR-reaction in *pBASTA-GUS* (Rottmann et al., 2016) yielding plasmid *pFC2*. Constructs for p*AtSUC5*:At*SUC5*g and pAt*SUC8*:At*SUC8*g reporter gene lines were also cloned in two fragments. For *AtSUC5* the first part was amplified with AtSUC5-2118fw+CACC and AtSUC5g+93rev, the second part with AtSUC5g+43fw and AtSUC5g+2079rev+A+AscI (**Supplementary Table 8**). Both fragments were combined in *pENTR/D-TOPO* (Invitrogen) *via* an internal *Bgl*II and an attached *Asc*I site. The complete construct was inserted into *pBASTA-GUS* (Rottmann et al., 2016) by LR, yielding plasmid *pFC5*. For *AtSUC8* the first part was amplified with AtSUC8-1544fw+CACC and AtSUC8g+238rev, the second part with AtSUC8g+191fw and AtSUC8g+1709rev+A+AscI (**Supplementary Table 8**). Both fragments were combined in *pENTR/D-TOPO* (Invitrogen) *via* an internal *Nde*I and an attached *Asc*I site. The complete construct was inserted into *pBASTA-GUS* (Rottmann et al., 2016) by LR, yielding plasmid *pFC8*. For generating *AtSUC4* reporter gene lines p*AtSUC4*:*AtSUC4*g insert was amplified with the primer pair AtSUC4-2044fw+CACC and AtSUC4g+2458rev (**Supplementary Table 8**). The p*AtSUC4*:*AtSUC4*g fragment was inserted into *pENTR/D-TOPO* (Invitrogen). By LR reaction the complete p*AtSUC4*:*AtSUC4*g construct were inserted into *pBASTA-GUS* (Rottmann et al., 2016), yielding the plasmid *pJS32* (p*AtSUC4*:*AtSUC4*g-*GUS*). For generating *AtSUC9* reporter gene lines p*AtSUC9*:*AtSUC9*g insert was amplified with the primer pair AtSUC9-2159fw+CACC and AtSUC9g+1926rev (**Supplementary Table 8**). The p*AtSUC9*:*AtSUC9*g fragment was inserted into *pENTR/D-TOPO* (Invitrogen). By LR reaction the complete p*AtSUC9*:*AtSUC9*g construct was inserted into *pBASTA-GUS* or p*BASTA-GFP* (Rottmann et al., 2016), yielding the plasmids *pTR319* (p*AtSUC9*:*AtSUC9*g-*GUS*) and *pTR320* (p*AtSUC9*:*AtSUC9*g-*GFP*). For cloning p*AtcwINV : AtcwINV*g, p*AtVI1*:*AtVI1*g-*GUS*, and p*AtVI2*:*AtVI2*g-*GUS* reporter gene lines the respective primer pairs used for amplification of the fragments including the promoter region and the complete genomic sequence are listed in **Supplementary Table 9**. For *AtcwINV1* an attached *NcoI* site, for *AtcwINV5* an attached *BamHI* site, and for *AtVI1* an attached *Asc*I site was used to ligate the respective fragments. The complete p*AtcwINV : AtcwINV*g and p*AtVI : AtVI*g-*GUS* constructs were respectively inserted into the destination vector *pBASTA-GUS* (Rottmann et al., 2016) by LR, resulting in the expression vectors *pJS1* (p*AtcwINV1*:*AtcwINV1*g-*GUS*), *pJS13* (p*AtcwINV2*:*AtcwINV2*g-*GUS*), *pJS14* (p*AtcwINV4*:*AtcwINV4*g-*GUS*) *pAL30* (p*AtcwINV5*:*AtcwINV5*g-*GUS*), *pJS37* (p*AtVI1*:*AtVI1*g-*GUS)*, and *pJS11* (p*AtVI2*:*AtVI2*g-*GUS)*. The stable pollen tube marker line p*LAT52*:*GFP*- *pMDC123-NosT* (*pTR53*) (Rottmann et al., 2018a), where *GFP* is expressed under the pollen-specific promoter, was used for the pollen tube esculin uptake assays.

### Cloning of constructs for complementation lines

For generation of the construct for complementation line Compl. AtSUC1 (p*AtSUC1*:*AtSUC1*g in *Atsuc1*), the 2025 bp fragment of the *AtSUC1* promoter and the complete genomic sequence including introns were amplified using primer pair AtSUC1-2025fw+CACC and AtSUC1g+1764rev (**Supplementary Table 10**) and inserted into *pENTR/D-TOPO* (Invitrogen). By LR reaction the complete construct was inserted into the destination vector *pMDC99* (Curtis & Grossniklaus, 2003), yielding the expression vector *pJS90*. *Atsuc1* knockout plants were transformed with *pJS90 via* floral dip method (Clough & Bent, 1998). For generation of the construct for complementation line Compl. AtSUC9 (p*AtSUC1*:*AtSUC9*g in *Atsuc1*) the method of In-Fusion™ cloning (Zhu et al., 2007) was performed. The first fragment was amplified from the vector p*AtSUC1*:*AtSUC1*g-*pENTR/D-TOPO* (*pJS79*) including the *pENTR/D-TOPO* vector backbone and the 2025 bp fragment of the *AtSUC1* promoter with the primer pair InFu1AtSUC1p(V)fw and InFu2AtSUC1p(V)rev (**Supplementary Table 10**). The second fragment was amplified from the vector p*AtSUC9*:*AtSUC9*g-*pENTR/D-TOPO* including the complete genomic sequence of *AtSUC9* including introns with the primer pair InFu3AtSUC9g(In)fw and InFu4AtSUC9g(In)rev. The specific primers were designed with the In-Fusion™ Primer Design Tool (https://www.takarabio.com/learning-centers/cloning/primer-design-and-other-tools). The PCR reactions were performed with PCRBIO VeriFi™ Mix Red (PCRBIOSYSTEMS) because of the big fragment size. The two fragments were united *via* the In-Fusion™ reaction with same amount of DNA of the respective two fragments for 15 min at 50°C, yielding the construct p*AtSUC1*p:*AtSUC9*g-*pENTR/D-TOPO*. By LR reaction the complete construct p*AtSUC1*:*AtSUC9*g was inserted into the destination vector *pMDC99* (Curtis & Grossniklaus, 2003), yielding the plasmid *pJS80* (see also **Table S7**). *Atsuc1* knockout plants were transformed with *pJS80 via* floral dip method (Clough and Bent, 1998). Pollen germination and pollen tube growth tests were analyzed with the T_2_ generation of the selected plants.

### Analyses of pollen germination and pollen tube growth

*In vitro* pollen germination and pollen tube growth tests with *Arabidopsis thaliana* pollen for RNA extraction and growth analyses were done as described (Rodriguez-Enriquez et al., 2013). The standard sucrose concentration was 250 mM within the medium. Different sucrose concentrations were indicated. Pollen germination rates were counted using ImageJ 1.53k (Schneider et al., 2012b). For length measurements, pollen tubes were usually allowed to grow for at least 7 – 8 h, unless indicated otherwise. Pollen tube lengths were measured with Smart Root (Lobet et al., 2011), a plugin of ImageJ, or with a self-written Python script (Python Software Foundation, Beaverton, OR, United States), and plotted with Excel (Microsoft 365). Pollen tube growth experiments were performed in three biological replicates per genotype and sucrose concentration. For pollen germination rates at least 500 pollen per each replicate, genotype, and sucrose concentration were counted and for pollen tube lengths at least 200 pollen tubes per each replicate, genotype and sucrose concentration were measured. Statistical analyses were performed in Excel (Microsoft 365) with *Anova* (single factor). The semi-*in vivo* pollen tube growth assays for analyses of reporter genes and RT-PCRs were also analyzed on medium described by Rodriguez-Enriquez et al. (2013), which were performed by pollinating stigmata by hand, cutting them off and placing them horizontally onto the cellulosic membrane of the growth medium to allow the outgrowth of the pollen tubes from the cut surface (Palanivelu & Preuss, 2006) for 4 – 6 h. For the pollen tube esculin uptake assays the semi-*in vivo* pollen tube growth assays of pollen tubes expressing *GFP* under the control of a pollen-specific promoter (*LAT52*) (Rottmann et al., 2018b) were analyzed on liquid pollen germination medium without cellulosic membrane with different pH values (5; 5.5; 6; 7; 9) containing 250 mM sucrose.

### Ion chromatographic measurements and starch staining

Pollen from different species were collected with the help of a vacuum cleaner with various tubes and filter nets with different pore sizes as described in Johnson-Brousseau and McCormick (2004). For ion chromatographic measurements the pollen were incubated with 300 µl 80% ethanol for 1 h at 80°C under constant shaking. Samples were centrifuged for 5 min at 14000 rpm at 4°C. As much supernatant as possible was transferred into a new Eppendorf tube (255 – 290 µl) and evaporated at 45°C in the Speed Vac. The pellet was resuspended in 125 µl sterile water by shaking at room temperature. The samples were centrifuged through a filter plate and pipetted in the respective tubes. The ion chromatographic measurements were done as described (Schneider et al., 2008). For starch staining collected pollen were stained with iodine potassium iodide solution while shaking for 4 – 5 h at 40°C.

### Pollen tube esculin uptake assay

Pollen tubes, which expressed *GFP* under the control of the pollen specific *LAT52* promoter, were grown semi-*in vivo* on liquid pollen germination medium with 250 mM sucrose and different pH values (5; 5.5; 6; 7; 9). To this end, 500 µl liquid germination medium was placed on a slide with recess and the pollinated stigmata, which were cut off, were placed horizontally onto liquid medium to allow the outgrowth of the pollen tubes from the cut surface for 3 – 4 h. Esculin in liquid growth medium was added to 500 µl liquid growth medium to a final concentration of 1 mM. After gently mixing esculin into the liquid growth medium, the slide was incubated for 40 min at room temperature at dark. Prior to microscopy the stigmata with the pollen tubes were gently transferred onto a slide with recess filled with liquid growth medium without esculin.

### Microscopy

Pictures of GUS plants were taken with a Leica MZFLIII stereomicroscope (Leica Microsystems) or with a Zeiss Axioskop (Carl Zeiss Jena GmbH). Pollen tubes for length measurements were analyzed by light microscopy (Zeiss Axioskop; Carl Zeiss Jena). Pollen stained with iodine potassium iodide solution were analyzed in bright field at 1000x magnification with a Zeiss Axioskop (Carl Zeiss Jena GmbH). Images of GFP-reporter plants and GFP expressing pollen tubes were taken on a Leica 765 TCS SPII confocal laser scanning microscope (Leica Microsystems) and processed with Leica Confocal Software 2.5. A 488-nm argon laser was used for excitation of GFP and chlorophyll autofluorescence. The 415-nm diode was used for the excitation of esculin fluorescence. Detection windows ranged from 497 to 526 nm for GFP, from 682 to 730 nm for chlorophyll autofluorescence and from 424 to 469 nm for esculin. Images of GFP and esculin fluorescence for the pollen tube esculin uptake assay were taken in a sequential mode. Image processing was done using analySIS Doku 3.2 software (Soft Imaging System, Münster) and GIMP2.10 (https://www.gimp.org/).

## Results

### Expression of sucrose transporter genes in reproductive organs

**Figure 1:**
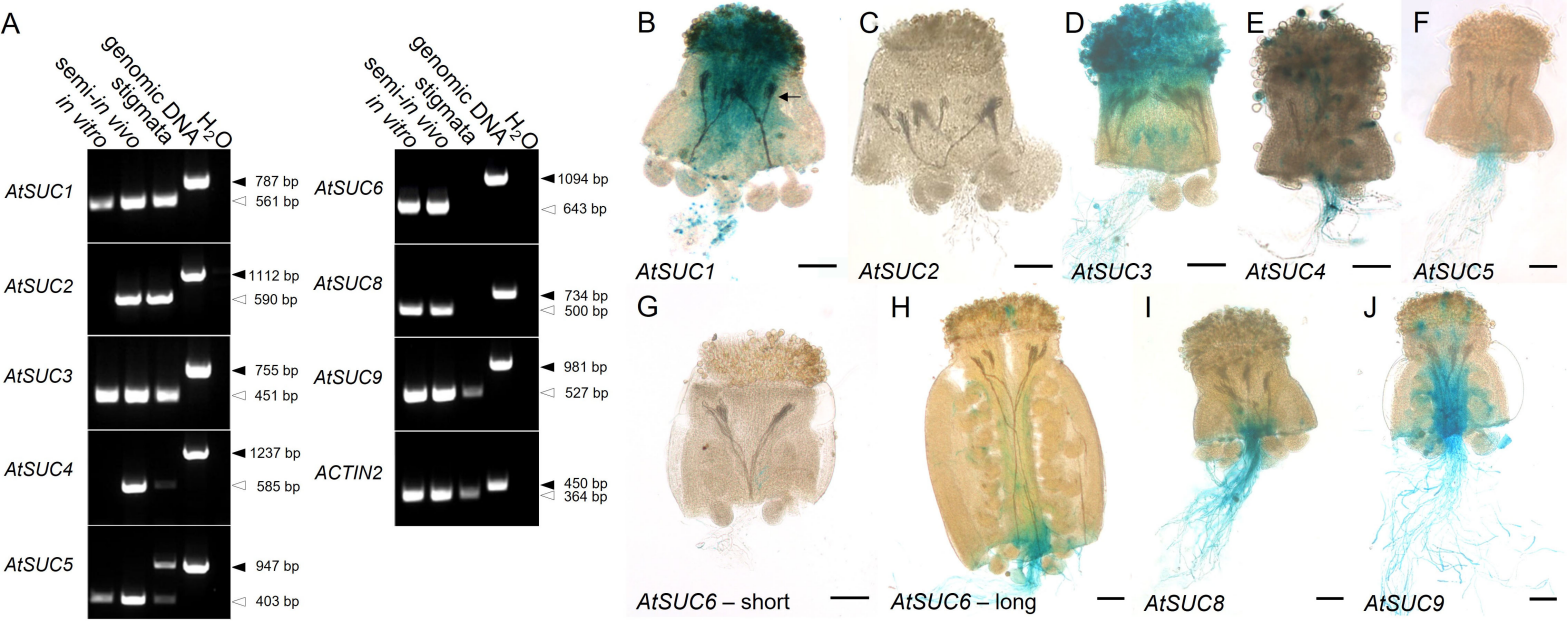
Analysis of *AtSUC* expression in pollen tubes. **(A)** RT-PCRs to study *AtSUC1* – *AtSUC9* expression in pollen tubes grown *in vitro* or in the upper part of a pollinated pistil (semi-*in vivo*) or in the upper part of an un-pollinated pistil (primers are listed in **Supplementary Table 3**). *AtSUC7* was not analyzed because it doesn`t encode a functional sucrose carrier. Arrows indicate the size of PCR products derived from reverse-transcribed mRNA (white) or from genomic DNA (black). The presence of cDNA in each sample was confirmed with *ACTIN2* specific primers (**Supplementary Table 3**). **(B–J)** Histochemical detection of β-glucuronidase activity in semi-*in vivo* growth assays with pollen tubes of p*AtSUC : AtSUC*g-*GUS*-reporter plants and stigmata from wild type plants. The arrow in **(B)** marks GUS containing pollen tubes growing through the WT style. For the expression pattern of *AtSUC2* p*AtSUC2*:*GUS* reporter plants were used. For AtSUC6, short **(G)** and long **(H)** parts of the pistil were used for the semi-*in vivo* assay. The analyzed genes are indicated in each figure. Tissues were stained with GUS solution for 48 h **(A)** or 2 - 5 h **(B–J)**. Scale bars: 100 µm in **(B–J)**.

RT-PCR and reporter gene analyses were performed to investigate the expression of sucrose transporter genes. Since it is known that gene expression in pollen can be induced after growth through stigma and style (Qin et al., 2009; Leydon et al., 2014), we performed semi-*in vivo* growth assays (see also **Figure 1B**). For this, pollinated stigmata were cut below the style and incubated on medium for seven hours to allow pollen tube growth. mRNA was extracted from *in vitro* grown pollen tubes as well as from the pollinated pistils-sections described above. The latter samples were labelled with “semi-*in vivo*”. They contained mRNA from pollen tubes and from the pistil. To distinguish between expression in the pistil and in pollen tubes, un-pollinated pistil-sections, labelled as “stigmata”, were used. *AtSUC2* is known to be companion cell specific and was used as a negative control for expression in pollen (Stadler and Sauer, 1996; Imlau et al., 1999; see also Stadler & Sauer, 2019). The RT-PCR data indicated that *AtSUC1*, *AtSUC3*, *AtSUC4*, *AtSUC5*, *AtSUC6*, *AtSUC8*, and *AtSUC9* were expressed in pollen tubes grown *in vitro* and in pollinated pistils (semi-*in vivo* samples, **Figure 1A**). *AtSUC7* was not analyzed because it is a pseudogene that does not code for an active sucrose transporter. *AtSUC2* is the only *AtSUC* gene, which was not expressed in pollen tubes. *AtSUC2* mRNA was only present in samples containing vascular tissue (**Figure 1A**). Since the pistil contains vascular bundles, both, the “stigmata” and the “semi-*in vivo*” sample showed a signal, although *AtSUC2* is not expressed in pollen. *AtSUC4* mRNA was detectable in pollen tubes grown semi-*in vivo*, but not in pollen tubes grown *in vitro*, indicating that the expression in pollen tubes was induced after growth through the stigma. For *AtSUC5* expression, the upper RT-PCR signal in the “stigmata” sample is caused by a contamination with genomic DNA, however, the assay clearly shows that the *AtSUC5* gene is expressed also in un-pollinated stigmata (**Figure 1A**). *AtSUC6* was expressed in pollen tubes grown *in vitro* and semi-*in vivo*, whereas no transcripts could be amplified from stigmata-derived cDNA. This is consistent with earlier results (Rottmann et al., 2018a). Besides *AtSUC2*, transcripts of *AtSUC1, AtSUC3*, *AtSUC4*, *AtSUC5*, and *AtSUC9* were also detectable in cDNA samples of un-pollinated pistil sections.

**Figure 2:**
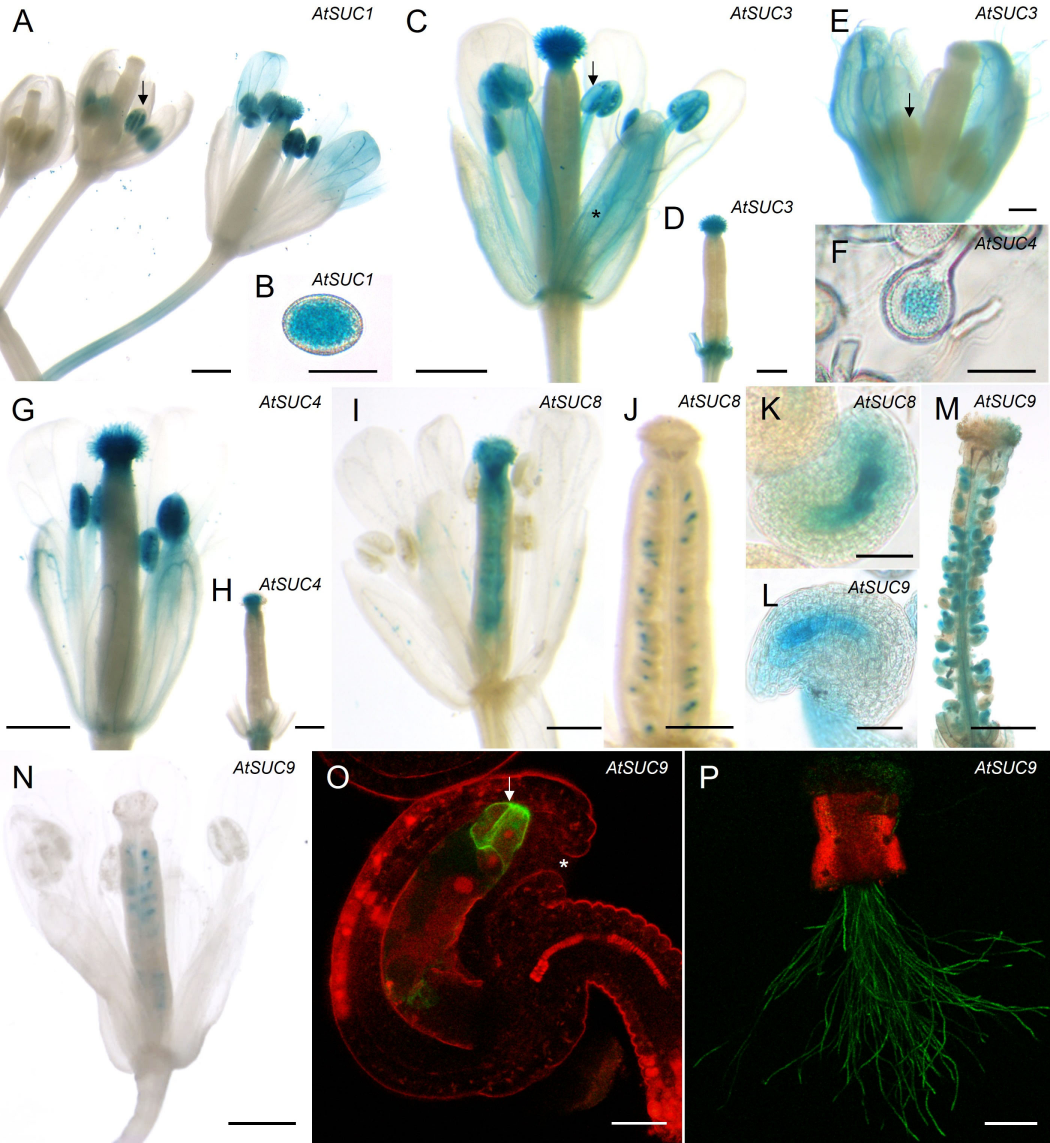
Analyses of *AtSUC* gene expression in reproductive organs. **(A–O)** Histochemical detection of ß-glucuronidase activity in *Arabidopsis thaliana* Col-0 expressing *AtSUC*g*-GUS* under the control of the native *AtSUC* promoter. *AtSUC2*, *AtSUC5* and *AtSUC6* expression was previously published, therefore the respective reporter lines were not included. **(A, B)** p*AtSUC1*:*AtSUC1*g-*GUS*. **(A)** Flower stage 10 (left), stage 12 (middle) and pollinated flower stage 14 (right). All floral stages according to Smyth et al., 1990 and Schneitz et al., 1995. **(B)** mature pollen. **(C, D, E)** p*AtSUC3*:*AtSUC3*g-*GUS*. **(C)** Pollinated flower of stage 14 (all flower stages according to Smyth et al., 1990). The asterisk marks blue stained vascular bundles. The arrow indicates blue mature pollen in an anther. **(D)** Un-pollinated pistil (stage 13) after removal of all other floral organs. **(E)** Flower stage 12, the arrow marks an un-stained anther. **(F–H)** p*AtSUC4*:*AtSUC4*g-*GUS*. **(F)** Pollen grains at higher magnification. **(G)** Pollinated flower stage 14. **(H)** Un-pollinated pistil (stage 13) after removal of other floral organs. **(I–K)** p*AtSUC8*:*AtSUC8*g-*GUS*. **(I)** Pollinated flower stage 14. **(J)** Un-pollinated ovary of stage 11. **(K)** Ovule at higher magnification. **(L–N)** p*AtSUC9:AtSUC9*g-*GUS*. **(L)** Ovule of an un-pollinated ovary at higher magnification. **(M)** Pollinated and peeled ovary of stage 14. **(N)** Pollinated flower of stage 14. **(O, P)** Detection of GFP fluorescence with a CLSM in *Arabidopsis thaliana* Col-0 expressing p*AtSUC9:AtSUC9*g*-GFP*. GFP fluorescence is shown in green, chlorophyll autofluorescence in red. **(O)** Ovule at higher magnification. The arrow marks GFP labelled synergid cells. The asterisk marks the micropyle. **(P)** Pollen tubes germinated semi-*in vivo* on a wild type (WT) stigma. Tissues were stained with GUS solution for 24 h **(A, B)** or 2 - 5 h **(C–N)**. Scale bars: 500 µm in **(A–E, H, I, L, M)**; 20 µm in **(F, G, O)**; 50 µm in **(J, K)**; 200 µm in **(N)**.

To analyze cell specific expression, we used existing or generated new *AtSUC* reporter gene lines, which included the endogenous promoter and the genomic sequence of the respective *AtSUC* genes. For *AtSUC2*, no new lines were generated since this gene is very well studied and is known to be companion cell specific and not expressed in pollen. Therefore, we used *AtSUC2p:GUS* lines which we had in the lab as a negative control. We performed semi-*in vivo* assays with pollen from GUS lines and WT pistils to investigate expression in pollen tubes after growth through stigma and style. The histochemical analyses confirmed the RT-PCR data and proved expression of *AtSUC1, AtSUC3, AtSUC4, AtSUC5, AtSUC6, AtSUC8*, and *AtSUC9* in pollen tubes (**Figures 1B–J**). Activity of AtSUC1-GUS fusion protein was well visible in parts of the pollen tubes that were growing through stigma and style (**Figure 1B**). In contrast, it was necessary to stain for 48 h to get a visible GUS signal in apical parts of pollen tubes of p*AtSUC1*:*AtSUC1*g*-GUS* plants emerging from WT pistils in *semi-in-vivo* assays (**Figure 1B**). This may point towards a major function of AtSUC1 during pollen germination and/or during early stages of pollen tube growth. The GUS stain in pictures of p*AtSUC1*:*AtSUC1*g*-GUS* assays had a dotted appearance, which might be due to the weak expression and/or to the long incubation in GUS staining solution. In contrast to *AtSUC1*, p*AtSUC6*:*AtSUC6*g*-GUS* expression increased during pollen tube growth through the transmitting tissue. Pollen tubes that emerged from long pistil parts were strongly stained (**Figure 1H**), which was also shown previously (Rottmann et al., 2018a), and growth through shorter pistil parts resulted in weak or almost invisible GUS signals (**Figure 1G**). The GUS analyses indicated that the sucrose carriers AtSUC1, AtSUC3 and AtSUC4 were already present in mature pollen in anthers, prior pollen germination on the stigma (**Figures 2A–G**). In addition, the pAtSUC1:AtSUC1g-GUS fusion protein was detectable in developing pollen during a later stage of anther development. pAtSUC1:AtSUC1g-GUS activity started in stage 12 flowers, which are characterized by papillar cells covering the stigma and a visible style (**Figure 2A**; floral stages according to Smyth et al., 1990 and Schneitz et al., 1995). *AtSUC3* was expressed in the vasculature of sepals and a strong expression was detectable in the stigma (**Figure 2C**). The *AtSUC3* expression in the stigma was not induced through pollination, as the expression was also visible in stigmata of un-pollinated pistils (**Figure 2D**). *AtSUC4* was expressed not only in growing pollen tubes but also in mature pollen in anthers and in cells of the stigma (**Figures 2F–H**). *AtSUC4* promoter activity in anthers of older and younger flowers has already been shown (Weise et al., 2000; Schneider et al., 2012a), but expression in the stigma is a novel finding. We carefully excluded cross-pollination during the experiments and analyzed 10 independent lines which all showed blue stigmata. A possible function of AtSUC4 activity in papillae cells could be the mobilization of vacuolar sucrose, either as a nutrient or for osmoregulation. Current analyses detected p*AtSUC8*:*AtSUC8*g*-GUS* expression not only in growing pollen tubes but also in ovules and in developing seeds (**Figures 2I–K**), which has not been observed during preceding studies (Sauer et al., 2004). Within ovules, the expression was restricted to the embryo sac (**Figure 2K**). We detected p*AtSUC9*:*AtSUC9*g*-GUS* expression in ovules and developing seeds (**Figures 2L–N**). Further investigations with p*AtSUC9*:*AtSUC9*g*-GFP* reporter gene lines showed an expression in synergid cells of ovules and confirmed the expression in pollen tubes (**Figures 2O, P**). In summary, the RT-PCR and reporter gene analyses showed that *AtSUC1* is expressed in developing pollen and that *AtSUC1*, *AtSUC3*, *AtSUC4*, *AtSUC5*, *AtSUC6*, *AtSUC8*, and *AtSUC9* are expressed in pollen tubes. In addition, *AtSUC3* and *AtSUC4* are expressed in stigmata and *AtSUC5*, *AtSUC8*, and *AtSUC9* in ovules.

### Characterization of *Atsuc* knockout lines

**Figure 3:**
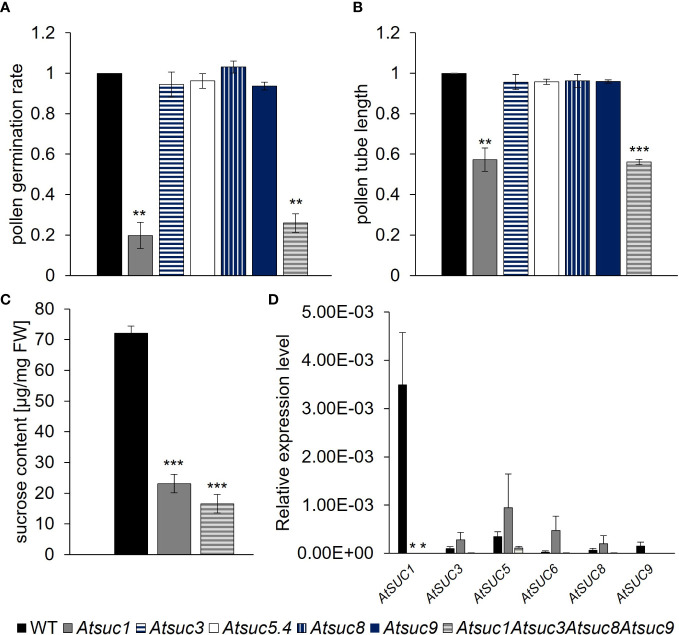
Germination, growth and sucrose content of pollen and *AtSUC* gene expression in stage 12 anthers of wild type and *Atsuc* knock out plants. **(A)** Pollen germination rates and **(B)** pollen tube lengths of wild type, *Atsuc3, Atsuc5.4, Atsuc8* and *Atsuc9* single and *Atsuc1,3,8,9* quadruple knock out plants after 7 h growth *in vitro* on medium with 250 mM sucrose. Bars represent mean values (+/- SE) of three biological replicates (pollen germination rate: n > 500, pollen tube lengths: n > 200 for each genotype in each experiment). **(C)** Ion chromatography measurements of sucrose content in pollen grains harvested from mature anthers of wild type, *Atsuc1* and *Atsuc1,3,8,9* knock out plants. Extracts of pollen of at least 72 plants per genotype were analyzed by ion chromatography and normalized to the fresh weight (FW) of the samples. **(D)** Relative expression levels of *AtSUC* genes in anthers of stage 12 flowers of WT (left bar), *Atsuc1* single knock out (middle) and *Atsuc1,3,8,9* quadruple knock out plants (right bar), quantified by RT-qPCR relative to *UBI10* expression. Primers are listed in **Supplementary Table 3**. Bars represent mean values of three biological replicates with three technical replicates each. The analyzed genes are indicated in each panel. For each biological replicate, the tissue for RNA isolation was collected from different plants. Significance = *Anova* **(A–C)** or Students *t*-test **(D)**, Reference: WT, *≙ p ≤ 0.05; **≙ p ≤ 0.01; ***≙ p ≤ 0.001.

Sivitz et al. published in 2008 that pollen of *Atsuc1* mutant lines had a significantly reduced pollen germination rate compared to wild type (WT), indicating a function for AtSUC1 in sucrose uptake into germinating pollen (Sivitz et al., 2008). To study the function of AtSUC1, AtSUC3, AtSUC5, AtSUC8, and AtSUC9 in pollen germination and pollen tube growth, single knockout lines were analyzed and compared with an *Atsuc1Atsuc3Atsuc8Atsuc9* quadruple knockout line, which was generated by multiple crossings of single knockout lines containing T-DNA insertions. Pollen of *Atsuc1* and *Atsuc1Atsuc3Atsuc8Atsuc9* mutant lines showed significantly reduced pollen germination rates and pollen tube lengths compared to wild type (**Figures 3A, B**). Thus, AtSUC1 is not only important for normal pollen germination, which was published previously, but also for pollen tube growth. Interestingly, the loss of three other sucrose transporters in addition to AtSUC1 did not cause a further reduction in germination or growth, and the single knockout plants *Atsuc3, Atsuc5.4, Atsuc8*, and *Atsuc9* did not show any defect regarding pollen germination and growth in comparison to WT (**Figures 3A, B**). These results demonstrate a major function of AtSUC1 during pollen tube growth and even more during pollen germination. To investigate the function of AtSUC proteins in sucrose import into developing pollen, we performed ion chromatography measurements of pollen that had been harvested from mature anthers. A high amount of sucrose was measurable within wild type pollen grains (70 µg/mg FW), which was reduced to less than 35% (~20 µg/mg FW) in *Atsuc1* mutant pollen (**Figure 3C**). Again, the loss of three other sucrose transporters in *Atsuc1Atsuc3Atsuc8Atsuc9* quadruple knockout pollen did not enhance the phenotype. This result points towards an important function of AtSUC1 in sucrose loading during pollen development. To analyze the presence of other sucrose carriers during late stages of pollen development, qRT-PCR analyses with mRNA samples from anthers of stage 12 flowers were performed. All *AtSUC* genes were analyzed except *AtSUC2, AtSUC4* and *AtSUC7* because AtSUC2 is companion cell – specific, AtSUC4 is localized in the tonoplast, and *AtSUC7* is a pseudogene. The data set resulted in a quite high standard error because stage 12 flowers are very small (1.5 mm long) and anthers are difficult to isolate in a sufficient amount. However, the analysis clearly showed that only *AtSUC1* mRNA was detectable in significant amounts in anthers of stage 12 flowers (**Figure 3D**). To test a potential complementation of the loss of *AtSUC1* by other *AtSUC* genes, single *Atsuc1* and quadruple *Atsuc1Atsuc3Atsuc8Atsuc9* knockout plants were included in the qRT-PCR analyses. An induction of other *AtSUC* genes in single and quadruple knockout anthers of stage 12 flowers could not be observed (**Figure 3D**). This also indicates a dominant role of AtSUC1 in sucrose import during late stages of pollen development.

### Complementation of the *Atsuc1* phenotype

**Figure 4:**
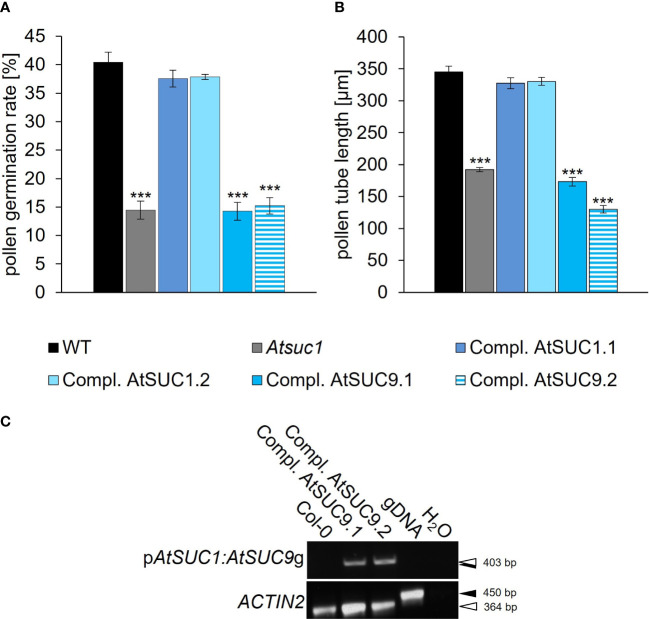
Complementation of the loss of *AtSUC1*. **(A)** Pollen germination rates and **(B)** pollen tube lengths of *Atsuc1* complementation lines compared to wild type pollen germinated *in vitro* on pollen germination medium containing 250 mM sucrose for 4 h. *Atsuc1* mutant plants expressing the genomic sequence of *AtSUC1* under the control of the native *AtSUC1* promoter (Compl. AtSUC1.1 + 1.2) or the genomic sequence of *AtSUC9* under the control of the native *AtSUC1* promoter (Compl. AtSUC9.1 + 9.2). Bars represent mean values (+/- SE) of three biological replicates (pollen germination rate: n > 500, pollen tube lengths: n > 200 for each genotype in each experiment). Significance: *Anova*. Reference: WT, **≙ p ≤ 0.01; ***≙ p ≤ 0.001. **(C)** RT-PCR analyses of *AtSUC1:AtSUC9*g expression in *in vitro* grown pollen tubes with primers specific for the *AtSUC1:AtSUC9* transgene including a forward primer that binds to the *AtSUC1* 5’ UTR and a reverse primer specific for *AtSUC9* (**Supplementary Table 5**). The resulting band doesn`t include expression of the intrinsic *AtSUC9* WT gene. The RT-PCR analyses were performed with mRNA isolated from pollen tubes of the respective complementation lines compared to wild type. Arrows indicate the size of PCR products derived from reverse-transcribed mRNA (white) and genomic DNA (gDNA; black). The presence of cDNA and gDNA in each sample was confirmed with *ACTIN2* specific primers.

To investigate the striking dominance in function of AtSUC1 among the other AtSUC proteins, we performed a complementation analysis. A construct containing the *AtSUC1* promoter and the *AtSUC9* genomic sequence was introduced into the *Atsuc1* background, and the resulting lines were compared with complementation lines containing the *AtSUC1* genomic sequence under the control of the *AtSUC1* promoter. The *AtSUC9* gene was chosen because it is known that AtSUC9 is correctly targeted to the plasma membrane in pollen (**Figure 2P**) and because of its similar *K*_M_ value (AtSUC1: 0.45 mM (Sauer & Stolz, 1994); AtSUC9: 0.5 mM (Sauer et al., 2004)). Pollen germination rates and pollen tube lengths of three different complementation lines homozygous for p*AtSUC1:AtSUC1*g in *Atsuc1* (Compl. AtSUC1.1 – 1.3) and eight different complementation lines expressing p*AtSUC1:AtSUC9*g in *Atsuc1* (Compl. AtSUC9.1 – 9.8) were analyzed and compared to wild type and *Atsuc1* (**Figures S1A, B**). Interestingly, the plants expressing p*AtSUC1:AtSUC9*g showed no rescued phenotype with pollen germination rates and tube lengths like those of *Atsuc1*, while pollen of *Atsuc1* plants expressing p*AtSUC1:AtSUC1*g were fully rescued and looked like wild type (**Figures 4A, B**, **S1A, B**). To examine if the hybrid construct of AtSUC1 promoter and AtSUC9 gene leads to gene expression, we performed RT-PCR analyses, which showed an expression of p*AtSUC1:AtSUC9*g in germinating pollen of the respective complementation lines (**Figure 4C**). The fact that the loss of only one out of six sucrose carriers that are present in pollen causes a severe phenotype and the observation that reintroduction of *AtSUC1* but not of *AtSUC9* restored the phenotype, indicate that AtSUC1 may play a special role during pollen germination and pollen tube growth that can`t be complemented by AtSUC9.

### Characterization of *Atsuc4.1* knockout mutants

Generally, sucrose can be stored in the vacuole or cytosol (Hedrich et al., 2015; Destailleur et al., 2021). *AtSUC4* encodes a sucrose carrier of the vacuolar membrane that exports sucrose from the vacuole into the cytoplasm. Our analysis showed that *AtSUC4* is expressed in pollen and pollen tubes (**Figures 2F, G**).

To address the function of AtSUC4 in sucrose mobilization from the vacuole, we analyzed pollen germination rates and pollen tube lengths of *Atsuc4.1* knockout plants (Schneider et al., 2012a) (**Figures S2A, B**). Furthermore, we studied a possible function of AtTMT1 and AtTMT2 which are known to catalyze the import of sucrose and glucose into the vacuole (Schulz et al., 2011). To this end, pollen of an *Attmt1Attmt2* (Schulz et al., 2011) double knockout line were analyzed (**Figures S2C, D**). Pollen germination rates and pollen tube lengths of *Atsuc4.1* and *Attmt1Attmt2* showed no significant difference compared to wild type (**Figure S2**). Obviously, neither AtSUC4 nor AtTMT1 and 2 have a major influence on pollen germination or pollen tube growth, at least *in vitro*. A possible explanation of this result would be that other transport proteins complement the loss of AtSUC4, AtTMT1, and AtTMT2, or that vacuolar invertases, which catalyze the cleavage of vacuolar sucrose into glucose and fructose, are involved in this process together with other monosaccharide transporters.

### Expression of cell wall and vacuolar invertase genes in reproductive organs

**Figure 5:**
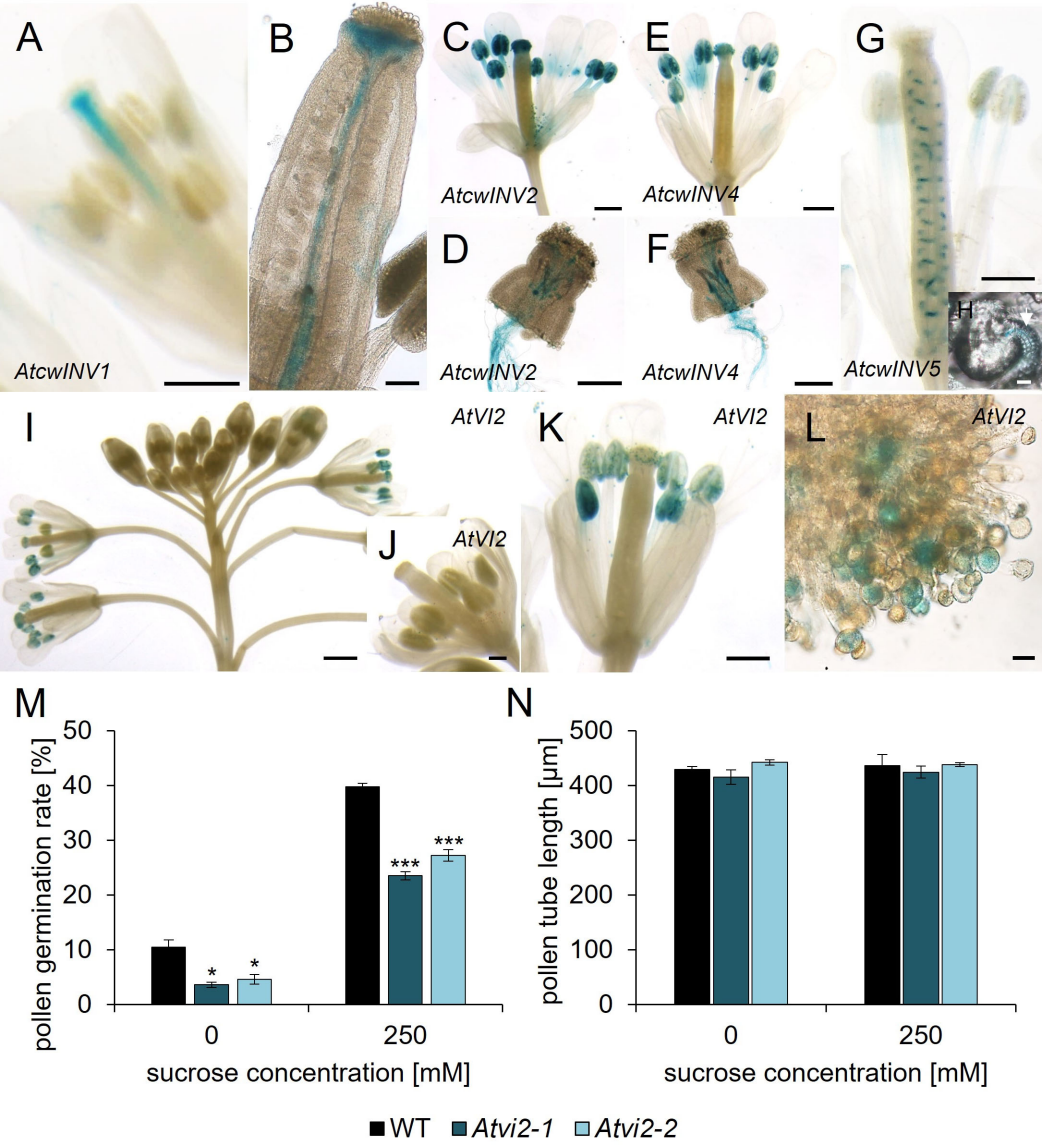
Analyses of p*AtcwINV : AtcwINV*g- and p*AtVI2*:*AtVI2*g-*GUS* reporter plants and *Atvi2* pollen germination and pollen tube growth tests. **(A–H)** Histochemical detection of ß-glucuronidase activity in *Arabidopsis thaliana* expressing *AtcwINV*g*-GUS* under the control of the native *AtcwINV* promoter. **(A)** stage 12 flower and **(B)** ovary of p*AtcwINV1:AtcwINV1*g-*GUS* plants. **(C, D)** p*AtcwINV2:AtcwINV2*g-*GUS.* **(C)** Pollinated flower stage 14. **(D)** p*AtcwINV2*:*AtcwINV2*g*-GUS* pollen tubes germinated semi-*in vivo* on a wild type stigma. **(E, F)** p*AtcwINV4:AtcwINV4*g-GUS. **(E)** Pollinated flower stage 14. **(F)** p*AtcwINV4*:*AtcwINV4*g*-GUS* pollen tubes germinated semi-*in vivo* on a wild type stigma. **(G, H)** p*AtcwINV5:AtcwINV5*g*-*GUS. **(G)** Pollinated flower stage 14. **(H)** p*AtcwINV5*:*AtcwINV5*g*-GUS* ovule with blue funiculi at higher magnification. **(I–L)** Histochemical detection of ß-glucuronidase activity in *Arabidopsis thaliana* expressing *AtVI2*g*-GUS* under the control of the native *AtVI2* promoter. **(I)** Inflorescence. **(J)** Flower stage 11. **(K)** Pollinated flower stage 14. **(L)** Pollen grains on a stigma at higher magnification. **(M)** Pollen germination rates and **(N)** pollen tube lengths of *Atvi2-1, Atvi2-2* and WT pollen germinated *in vitro* for 7 h on pollen germination medium with different sucrose concentrations. Bars represent mean values (+/- SE) of three biological replicates (pollen germination rate: n > 500, pollen tube lengths: n > 200 for each genotype in each experiment). Significance: *Anova*. Reference: WT, *≙ p ≤ 0.05; ***≙ p ≤ 0.001. Tissues were stained with GUS solution for 22 - 24 h **(A – F)** or 48 h **(G, H)** or 24 - 44 h **(I–L)**. Scale bars: 500 µm in **(A, C, E, G, K)**; 200 µm in **(D, F, J)**; 100 µm in **(B)**; 1 mm in **(I)**; 20 µm in **(H, L)**.

To investigate a potential function of invertases during pollen tube growth we first analyzed the expression pattern of the respective invertase genes. Reporter gene lines were generated for the cell wall and vacuolar invertase genes, except *AtcwINV3* and *AtcwINV6*, containing the genomic sequence of the respective *AtcwINV* or *AtVI* gene with its native promoter in front of the *GUS* gene (**Figure 5**). p*AtcwINV1*:*AtcwINV1*g*-GUS* showed an expression within the transmitting tissue (**Figures 5A, B**). The genes of the cell wall invertases *AtcwINV2* and *AtcwINV4* were expressed in pollen and pollen tubes (**Figures 5C–F**). The p*AtcwINV5*:*AtcwINV5*g *-GUS* fusion protein was detectable in filaments of stamens and in funiculi of the pistil (**Figures 5G, H**). There was no *AtVI1* expression detectable in flowers or pollen tubes (**Figure S5**), however, p*AtVI2*:*AtVI2*g*-GUS* showed expression in mature pollen (**Figures 5I–L**). Blue GUS staining was detectable in anthers as soon as petals and filaments had elongated, while no blue staining was visible during earlier stages of flower development. In closed flowers there was no *AtVI2*-expression detectable at all (**Figures 5I, J**). Pollen from p*AtVI2*:*AtVI2*g *-GUS* applied to a wild type stigma showed *AtVI2* expression (**Figure 5L**). Thus, *AtVI2* is the only vacuolar invertase expressed in mature pollen.

### Characterization of *Atcwinv* and *Atvi2* knockout lines

To monitor invertase function during pollen tube growth we generated a triple knockout mutant with T-DNA insertions in *AtcwINV2, AtcwINV4* and *AtcwINV5*. The generation of a quadruple knockout line was not possible because of the linkage of the genes *AtcwINV1* and *AtcwINV5*. Furthermore, *AtcwINV1* is not expressed in pollen. Pollen germination rates and pollen tube lengths of *Atcwinv2-1Atcwinv4-2Atcwinv5* plants showed no difference compared to wild type (**Figures S4A, B**). Two *Atvi2* knockout mutant lines, *Atvi2-1* (SALK_011312) and *Atvi2-2* (SALK_100813) (**Figure S3**) were characterized in pollen germination assays. Both lines showed significant reduced *in vitro* pollen germination to almost 50% compared to wild type pollen on germination medium with 0 or 250 mM sucrose (**Figure 5M**). The addition of sucrose to the external medium didn`t rescue the reduced germination rate of *Atvi2* pollen. Since two independent *Atvi2* T-DNA insertion lines showed a similar reduction in pollen germination, it can be concluded that the effect is caused by the loss of AtVI2 function. However, the few *Atvi2* knockout pollen, which germinated, developed a similar pollen tube length as wild type pollen on medium without or with 250 mM sucrose (**Figure 5N**). In summary, the results indicated that the vacuolar invertase AtVI2 is important for efficient pollen germination at least *in vitro*, while there is no influence of cell wall invertases on pollen germination or pollen tube growth.

### Carbohydrate storage in mature pollen and further analyses of pollen tube growth

**Figure 6:**
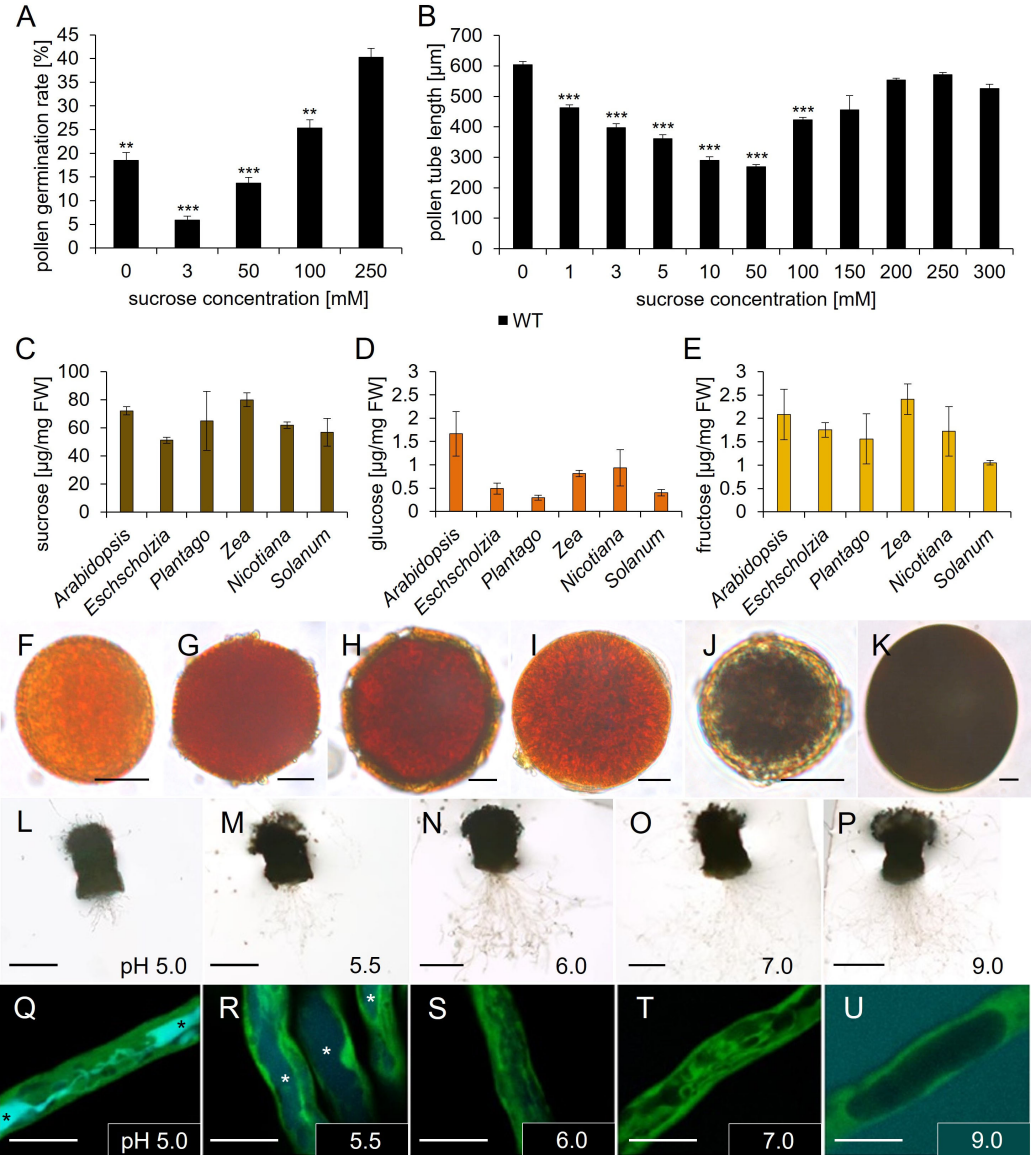
Analyses of *Arabidopsis thaliana* pollen tube growth, sugar, and starch content of pollen grains of different species. **(A)** Pollen germination rates and **(B)** pollen tube lengths of pollen grown on germination medium with different sucrose concentrations for 7 h. Mean values +/- SE of three biological replicates are shown (germination rate: n > 500, pollen tube length: n > 250 for each sucrose concentration). **(C–E)** Ion chromatography measurements of **(C)** sucrose, **(D)** glucose, and **(E)** fructose contents of pollen from different species. *Arabidopsis* = *A. thaliana*; *Eschscholzia* = *E. californica; Plantago* = *P. major; Zea* = *Z. mays*; *Nicotiana* = *N. tabacum*; *Solanum* = *S. lycopersicum*. Mean values +/- SE of three biological replicates are shown. **(F–K)** Starch staining of pollen grains from different species with iodine potassium iodide solution. **(F)** *Arabidopsis thaliana*. **(G)** *Coleus blumei*. **(H)** *Eschscholzia californica*. **(I)** *Phaseolus* spec. **(J)** *Plantago major*. **(K)** *Zea mays*. **(L–U)** Esculin uptake of GFP pollen tubes, which were grown semi-*in vivo* on liquid pollen germination medium with 250 mM sucrose and different pH values for 6h, were incubated with 1 mM esculin in pollen germination medium for 40 min. Asterisks mark vacuoles containing esculin. **(L–P)** Pollinated stigmata with growing pollen tubes on medium with indicated pH values. **(Q–U)** Visualization of esculin uptake into individual pollen tubes. GFP fluorescence is shown in green and esculin fluorescence in cyan. Significance: *Anova*. Reference: WT, *≙ p ≤ 0.05; **≙ p ≤ 0.01; ***≙ p ≤ 0.001. Scale bars: 10 µm in (**F–K**; **Q–U**); 500 µm in **(L–P)**.

Pollen express several genes for sucrose transporters, however, it is unknown if there is sucrose available in the apoplasm of the stigma and the transmitting tissue. Since it is difficult to examine the amount of apoplasmic sucrose *in vivo* we investigated the impact of apoplasmic sucrose on pollen germination and pollen tube growth *in vitro.* The analyses showed that pollen germination was reduced by 50% on medium without any sucrose compared to medium with 250 mM sucrose (**Figure 6A**). This observation indicated that sucrose promotes germination of wild type pollen. Interestingly, on medium containing 3 mM sucrose, the pollen germination rate was even worse than on medium without sucrose. The addition of 100 mM or 250 mM sucrose led to a steady increase in pollen germination rate (**Figure 6A**). These measurements were highly reproducible and independent from pollen batches or season. In contrast to pollen germination tests, pollen tube growth tests demonstrated no differences in pollen tube length on medium containing 250 mM sucrose compared to medium containing no sucrose (**Figure 6B**). However, the supplement of 1 mM up to 50 mM sucrose caused a progressive reduction in pollen tube length with a minimal length at 50 mM sucrose. Concentrations higher than 50 mM promoted pollen tube growth with a peak in a concentration range of 200 mM to 300 mM sucrose. In summary, it can be concluded that the presence of sucrose promotes pollen germination, while sucrose seems to be not necessary for pollen tube growth, because pollen tubes grew very long on medium without sucrose. Since the pollen tube growth medium (Rodriguez-Enriquez et al., 2013) didn`t contain any other carbohydrate, lipid or even protein, which could be metabolized, this result points towards internal energy reserves. To analyze this, we performed sugar measurements and starch staining assays of pollen from *Arabidopsis thaliana*. For comparison purposes, we included pollen from several unrelated plant species, *Eschscholzia californica*, *Plantago major*, *Zea mays*, *Nicotiana tabacum* and *Solanum lycopersicum*. Ion chromatography measurements observed high contents of sucrose (50 – 80 µg/mg FW) within pollen of all investigated species, compared to low glucose (1 – 2 µg/mg FW) and fructose (1 – 2.5 µg/mg FW) levels (**Figures 6C–E**). Pollen grains of *Arabidopsis thaliana* and *Zea mays* contained the highest amount of sucrose. Interestingly, the ratio between glucose and fructose varied between species. *Arabidopsis thaliana* pollen accumulated similar amounts of glucose and fructose, while pollen of the other species stored 2 – 3 times more fructose (**Figures 6D, E**). This could be due to different substrate specificities of transport proteins that accumulate monosaccharides during pollen development or to different activities of sucrolytic enzymes and downstream metabolic pathways. The glucose-to-fructose ratio was shown to influence pollen tube growth in a species specific manner (Rottmann et al., 2018b). To analyze starch storages, we performed iodine based staining assays (**Figures 6F–K**). Pollen grains from *Plantago major* (*Plantaginaceae*) and *Zea mays* (*Poaceae*) showed a dark staining, indicating a high amount of starch (**Figures 6J, K**). All other pollen from *Arabidopsis thaliana* (*Brassicaceae*), *Coleus blumei (Lamiaceae), Eschscholzia californica (Papaveraceae)* and *Phaseolus* spec. *(Fabaceae)* showed no dark staining, indicating that there is no starch or the starch content within the pollen of these species is too low for detection with iodine potassium iodide solution (**Figures 6F–I**). Another approach was to investigate if the pH value interferes with sucrose uptake or pollen tube growth. The activity of sucrose importers can be experimentally visualized by the uptake of esculin, a fluorescent sucrose analogue for AtSUC/AtSUT and AtSWEET proteins. Under conditions in which carriers are active, the fluorescent dye is transported into the cytosol and from there by an unknown transport protein into the vacuole, where it accumulates (Rottmann et al., 2018a). Pollen of a transgenic line expressing *GFP* under the control of a pollen tube specific promoter *LAT52* (Rottmann et al., 2018b) were used for semi-*in vivo* growth assays on liquid medium with different pH values. Esculin uptake was very efficient at pH 5.0 resulting in bright cyan fluorescence in the vacuole (**Figure 6Q**). On medium with pH 5.5, vacuolar esculin fluorescence was weaker but still visible (**Figure 6R**). At pH 6, pH 7, and pH 9 no esculin fluorescence was visible in pollen tubes anymore, which indicates that the sucrose carriers are not active under these conditions (**Figures 6S–U**). Interestingly, the opposite effect of the external pH could be observed regarding pollen tube growth semi-*in vivo*: the best growth was visible on medium with pH values from 6.0 to 9.0, while pollen tube growth seemed to be inhibited on pH 5.0 and weaker also on pH 5.5 (**Figures 6L–P**).

## Discussion

### Seven genes encoding sucrose carriers are expressed in pollen and/or pollen tubes of *Arabidopsis thaliana*

Initial studies on *AtSUC* gene expression often used reporter gene lines that expressed *GUS* or *GFP* coupled to the respective promoter without the genomic sequence of the gene (Truernit and Sauer, 1995 *AtSUC2*; Imlau et al., 1999 *AtSUC2*; Stadler et al., 1999 *AtSUC1*; Meyer et al., 2000; Meyer et al., 2004 *AtSUC3*; Baud et al., 2005 *AtSUC5*). Several recent publications indicated an influence of intron sequences on the expression pattern of sugar transporter genes (Rose, 2004; Sivitz et al., 2007; Weise et al., 2008; Rottmann et al., 2018a; Lasin et al., 2020). We generated reporter gene lines that expressed p*AtSUC : AtSUC*g-*GUS* fusion constructs including intragenic sequences to analyze the expression pattern of genes encoding sucrose carriers in pollen and/or pollen tubes. The reporter gene lines demonstrated the presence of AtSUC1, AtSUC3, AtSUC4, AtSUC5, AtSUC6, AtSUC8, and AtSUC9 in the male gametophyte. Expression of *AtSUC1*, *AtSUC3*, and *AtSUC6* in pollen has already been published (Stadler et al., 1999; Meyer et al., 2000; Meyer et al., 2004; Sivitz et al., 2008; Rottmann et al., 2018a). Together with earlier publications our data indicate that the phloem specific *AtSUC2* is the only gene of the *SUC/SUT* gene family in *Arabidopsis thaliana*, which is not expressed in pollen. All other *AtSUC* genes of the Arabidopsis genome are expressed in the male gametophyte during pollen tube growth towards the ovule, including the pseudogene *AtSUC7* (Rottmann et al., 2018a). Among the AtSUC proteins only AtSUC4 resides in the vacuolar membrane, where it could be essential to mobilize carbohydrates from vacuolar storages (Schneider et al., 2012a). Uptake of glucose and sucrose into the vacuole could be mediated by AtTMT1 and AtTMT2 (Schulz et al., 2011). However, loss of either AtSUC4 or AtTMT1 and AtTMT2 had no influence on pollen germination and tube growth, indicating that neither import *via* AtTMT1 or AtTMT2, nor export of sucrose from the vacuole *via* AtSUC4 is necessary for pollen germination or pollen tube growth. There may be other transporters that import sucrose into the vacuole and compensate for the missing AtTMTs, and AtSUC4 could be replaced by vacuolar invertases and monosaccharide transport proteins in the tonoplast. Indeed, we could show that the vacuolar invertase AtVI2 is important for pollen germination as discussed below.

All other AtSUC proteins are targeted to the plasma membrane. Why are so many transport proteins present in the plasma membrane of pollen tubes? To address this question, we analyzed knockout mutants with T-DNA insertions in *AtSUC1*, *AtSUC3*, *AtSUC8*, or *AtSUC9*. Sivitz and coworkers showed that AtSUC1 loss of function mutants are less fertile and the pollen germination rate is decreased (Sivitz et al., 2008). We confirmed this observation and found in addition, that *Atsuc1* pollen tubes grew also significantly shorter *in vitro*. This indicates that AtSUC1 is not only important for pollen germination, but also for pollen tube growth. Interestingly, except *Atsuc1*, none of the abovementioned single knockout lines developed pollen with altered germination rate or altered pollen tube length *in vitro*. Similarly, *Atsuc5* and *Atsuc6* knockout lines produced normal pollen (Baud et al., 2005; Rottmann et al., 2018a). *Atsuc1Atsuc3Atsuc8Atsuc9* quadruple knockout pollen had a similar phenotype compared to *Atsuc1* single knockout pollen. However, it is still possible, that additional loss of AtSUC5 and/or AtSUC6 would enhance this phenotype. Moreover, expression of *AtSUC9* under the control of the *AtSUC1* promoter was not able to complement the *Atsuc1* pollen phenotype. AtSUC9 belongs to the same clade as AtSUC1 and shares a sequence similarity of 85.4% (Sauer et al., 2004). The substrate specificity for sucrose uptake as well as the *K*_M_ values for sucrose are very similar among the AtSUC proteins in *Arabidopsis thaliana* (Sauer, 2007). The *K*_M_ values for sucrose estimated by heterologous expression in baker’s yeast were 0.45 mM for AtSUC1 and 0.5 mM for AtSUC9 (Sauer & Stolz, 1994; Sauer et al., 2004). A higher sucrose affinity as well as a high transport activity also in neutral pH ranges had been measured for AtSUC9 in oocytes (Sivitz et al., 2007). However, these differences alone probably are not sufficient to explain the missing ability of AtSUC9 to complement the loss of AtSUC1. Although heterologous expression systems didn`t demonstrate severe functional differences, a change of 15% of the amino acids could of course be responsible for discrepancies in substrate specificity, Km values or protein modification and regulation of the sucrose carriers in pollen. Generally, AtSUC1 may go through posttranslational modification events that affect its activity, or AtSUC1 could have an additional function maybe not only as sucrose transport protein. It could work as a protein that is interfering with hormonal pathways or other signal transduction events important for pollen tube growth, or as a sucrose sensor that controls carbohydrate metabolism or other events that regulate pollen germination or pollen tube growth.

Besides a difference in protein function, another possibility would be that AtSUC9 isn`t targeted correctly to the plasma membrane. However, translational reporter gene fusions showed that all *AtSUC* genes except *AtSUC2* are transcribed and translated in pollen, and for pAtSUC9:AtSUC9g-GFP we could prove that the protein is correctly targeted to the plasma membrane in pollen tubes (Rottmann et al., 2018a). Differences in transcriptional regulation could also be an explanation for the failure of p*AtSUC1:AtSUC9*g complementation construct to rescue the *Atsuc1* knock out phenotype. As shown recently, the *AtSUC1* introns act as strong enhancer for expression in roots, although they are not necessary to drive expression in mature pollen (Lasin et al., 2020). It still may be possible that the introns of *AtSUC1* are necessary for *AtSUC1* transcription in developing pollen.

### AtSUC1 is essential for preloading pollen with sucrose

**Figure 7:**
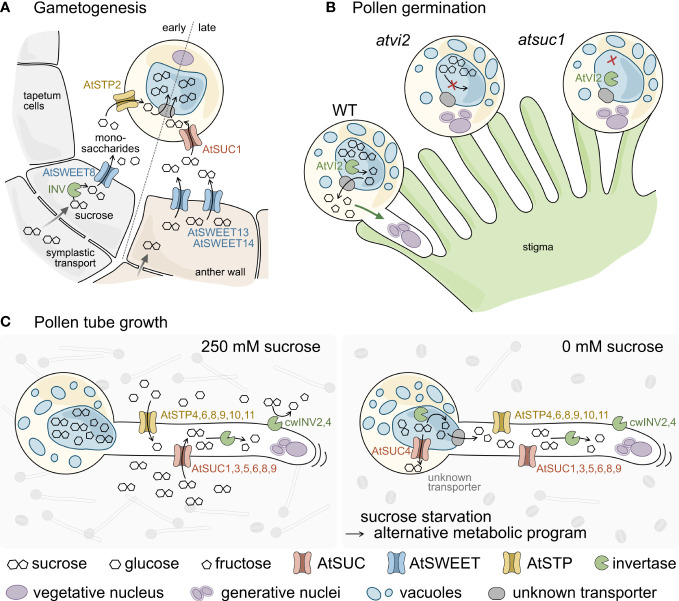
Schematic model of sugar transport during pollen development, germination, and tube growth **(A–C)**. **(A)** Sucrose is delivered into cells of the anther wall from source tissues *via* symplasmic transport. During early gametogenesis sucrose is cleaved by intracellular invertases (INV) and unloaded into the locules through AtSWEET8. AtSTP2 then mediates uptake of the monosaccharides across the plasma membrane of the pollen. At late anther developmental stages sucrose from the anther wall is exported *via* AtSWEET13 and 14 and taken up into pollen through AtSUC1. The imported sugars are probably imported into the vacuole *via* so far unknown transporters (grey) and accumulate to extremely high concentrations. **(B)** Pollen germination on the stigmatic papillae requires AtVI2 to cleave the sucrose molecules stored in the vacuole. The resulting monosaccharides are exported to the cytosol *via* so far unknown transporters and promote pollen tube growth. Both, the lack of AtVI2 in *Atvi2* mutants as well as a reduced sucrose content in *Atsuc1* prevent successful pollen germination. **(C)** Under *in vitro* conditions pollen germinate and pollen tubes elongate best at apoplasmic sucrose concentrations of about 250 mM. Apoplasmic sucrose can either be imported directly *via* AtSUC1, 3, 5, 6, 8, and 9 or is cleaved by cell wall invertases (AtcwINV2 and 4). The resulting monosaccharides are taken up by a set of AtSTP monosaccharide transporters and the imported sugars provide energy for pollen tube elongation. Under sucrose starvation conditions (0 mM sucrose) pollen germination rates are much lower (sketched in the background). However, some pollen manage to germinate and grow long pollen tubes, indicating that the absence of sucrose in the apoplasm induces an alternative metabolic program. This probably involves the export of pre-stored sucrose from the vacuole *via* AtSUC4 or through unknown monosaccharide transporters after cleavage by vacuolar invertases.

Ion chromatographic measurements showed a significantly reduced sucrose content in mature pollen of *Atsuc1* plants compared to WT. The measurements showed that wild type pollen are already preloaded with high amounts of sucrose during maturation in the anthers (70 μg/mg FW). GUS assays and qRT-PCR analyses indicated that AtSUC1 is the only sucrose carrier that is present during pollen development in stage 12 flowers. We postulate an important function of AtSUC1 in building up a carbohydrate storage during pollen development. It was shown previously that *AtSUC1* mRNA is present in developing pollen (Stadler et al., 1999; Bock et al., 2006). However, the conclusion that the AtSUC1 protein is also present in developing pollen and plays a major role in this stage contradicts our own results from 1999 (Stadler et al., 1999). In this earlier publication the AtSUC1 protein was identified *in situ* with αAtSUC1 antibodies only in germinating pollen on the stigma, but not in mature pollen. However, here we clearly show that the pAtSUC1:AtSUC1g-GUS fusion protein is present in pollen of anthers of stage 12 flowers. We think that we may have overlooked this stage when we performed the immunological detection of AtSUC1 in anther cross sections (Stadler et al., 1999). The expression of AtSUC1 in stage 12 flowers exactly overlaps with the expression of *AtSWEET13* and *AtSWEET14*, which encode sucrose exporters that are essential to unload sucrose from the anther wall into the apoplasm (Wang et al., 2022). Pollen of *Atsweet13Atsweet14* double knockout mutants have a similar phenotype as pollen of *Atsuc1* knockout mutants, in terms of reduced germination rate and less sucrose content. We postulate that AtSUC1 is responsible for the import of sucrose, which had been exported by AtSWEET13 and AtSWEET14 in late stages of pollen development, maybe only during a short time period. Carbohydrate import into developing pollen may occur in two steps, an early and a late step, as suggested by Wang et al. (2022): During early stages of pollen development, AtSWEET8/RUPTURED POLLEN GRAIN1 releases monosaccharides from the tapetum and AtSTP2 catalyzes the uptake of glucose into pollen (Truernit et al., 1999; Guan et al., 2008). During late stages of pollen development, AtSUC1 functions together with AtSWEET13 and AtSWEET14 in building up a sucrose storage in pollen (see also **Figure 7**).

### Expression of *AtSUC* genes in other reproductive tissues

Beside their localization in pollen, the AtSUC proteins are present in other floral tissues. AtSUC3 was identified in stigmata which corresponds to the results from older publications on *AtSUC3* promoter activities (Barker et al., 2000; Meyer et al., 2000; Schulze et al., 2000; Schulze et al., 2003; Meyer et al., 2004). A sucrose transporter in the stigma could be important for nutrition as the stigma is a sink tissue with only a few chloroplasts in the papilla cells. Alternatively, the imported sucrose could serve as an osmotic driving force for the papilla cells to maintain the turgor, which is essential for their task as pollen catchers. According to earlier publications, the *AtSUC4* gene is expressed in roots, anthers, and vascular tissues of source leaves (Weise et al., 2000; Schulze et al., 2003; Schneider et al., 2012a). In addition, we detected an *AtSUC4* expression in the stigma and in germinating pollen. This expression pattern indicates a function of AtSUC4 in supplying carbohydrates, which have been stored in the vacuole, to stigmatic cells and to growing pollen tubes. AtSUC5 catalyzes the transport of sucrose and biotin into developing seeds and the gene is expressed primarily in developing embryos and seeds, but also in roots, leaves, flowers, and siliques (Ludwig et al., 2000; Baud et al., 2005; Pommerrenig et al., 2013). Here we could show that the *AtSUC5* expression is already present in ovules of un-pollinated flowers. In this early developmental stage, AtSUC5 is assumed to be responsible for sucrose loading into ovules and later the embryo to build up a carbohydrate storage for seedling growth. Earlier reporter plant studies without the intragenic regions of *AtSUC8* reported GUS activity only in the transmitting tissue of the ovary, which resembled GUS activity derived from pollen tubes growing through the pistil (Sauer et al., 2004). In our publication, reporter gene analyses with *AtSUC8* promoter and gene showed expression in the stigma and in the central cells of ovules. Previous *AtSUC9* expression studies showed ß-glucuronidase activity in flowers and embryos (Sauer et al., 2004; Sivitz et al., 2007). In the present work, an expression of p*AtSUC9*:*AtSUC9*g*-GUS* was additionally detected in synergids. AtSUC9-mediated sucrose import into synergids could be important to cover the energy demand of these cells.

### The vacuolar invertase AtVI2 is essential for efficient pollen germination

We also studied the expression of genes encoding cell wall and vacuolar invertases. We observed an expression of *AtcwINV1* in the transmitting tissue of the pistil, which is in line with RT-PCR analyses from Sherson and coworkers that detected the transcript in flowers (Sherson et al., 2003). However, *Atcwinv1* lines are fully fertile indicating that AtcwINV1 has no major impact on pollen tube growth. For *AtcwINV2* and *AtcwINV4*, GUS reporter gene lines demonstrated expression in pollen and pollen tubes, which also coincides with transcript analyses of other groups (Sherson et al., 2003; Su et al., 2016; Li et al., 2021). Our reporter gene studies detected *AtcwINV5* expression in funiculi of the ovary. Again, the observations are in line with previous results (Sherson et al., 2003; Su et al., 2016). AtcwINV5 could provide hexoses for nutrition or as a signal for guidance of pollen tubes that grow *via* the funiculi to the ovules. Interestingly, pollen germination and pollen tube growth tests in this study with pollen of *Atcwinv2-1Atcwinv4-2Atcwinv5* triple mutant showed no difference to the wild type regarding both, germination rate and pollen tube length. This seemed to contradict previous results from Hirsche et al. (2009). The authors found that the expression of an RNA interference construct designed to recognize *AtcwINV2* mRNA led to greatly reduced pollen germination and seed production rates in *Arabidopsis thaliana* and *Nicotiana tabacum* (Hirsche et al., 2009). It is possible that the loss of three cell wall invertases in the triple knockout leads to an activation of other invertase genes like *AtcwINV1*, which could then complement for the loss. Another explanation for this discrepancy would be that RNA interference constructs could lead to the knockout of other related genes, maybe also of genes coding for vacuolar invertases, which could cause the observed effect. The functional coupling of sucrose cleavage by invertases and the uptake of the monosaccharides by monosaccharide transporters was shown to be crucial for pollination in tobacco (Goetz et al., 2017). Tobacco pollen from plants overexpressing the invertase inhibitor *NtCIF* displayed lower vitality and a reduced germination efficiency (Goetz et al., 2017). We can conclude from our data that the loss of cell wall invertases AtcwINV2, AtcwINV4, and AtcwINV5 has no major influence on the germination of pollen or pollen tube growth in Arabidopsis *in vitro*.

One of the two vacuolar invertase genes, *AtVI2*, is expressed in pollen, while GUS analyses showed that *AtVI1* is not expressed in pollen (**Figure S5**). Pollen from independent knockout lines *Atvi2-1* and *Atvi2-2* showed a significantly reduced germination rate *in vitro* on medium without and with 250 mM sucrose. On medium without sucrose, germination of *Atvi2* pollen was even more reduced. Obviously, cleavage of vacuolar sucrose is essential for normal pollen germination *in vitro*. The monosaccharides may be transported to the cytosol and serve as energy source for pollen germination or pollen tube growth. Addition of apoplasmic sucrose could not compensate for the missing sucrose breakdown in pollen of *Atvi2* knockout lines. Sucrose cleavage in the vacuole may be a signal that promotes pollen germination, and both, vacuolar as well as apoplasmic sucrose is necessary for germination *in vitro* (see also **Figure 7**). For pollen tube growth, however, AtVI2 does not seem to be important.

### Pollen need apoplasmic sucrose for germination although they are packed with sucrose

To get information on internal carbohydrate storages we performed iodine staining assays and detected no starch in pollen of *Arabidopsis thaliana*, while other species, especially *Zea mays* and *Plantago major*, showed a strong iodine-starch staining reaction. Consequently, starch does not seem to be present in high amounts, which is consistent with results from earlier publications (Kuang & Musgrave, 1996; Tang et al., 2009; Streb & Zeeman, 2012). Overall, some starch grains may be present within mature Arabidopsis pollen, however, it can be concluded that starch does not seem to be the major storage compound in pollen of this species. Ion chromatography measurements showed that pollen of Arabidopsis and all other species used in this study accumulate a very high amount of sucrose [50 – 80 µg/mg FW sucrose; for comparison: Arabidopsis roots contain 0.17 μg/mg FW sucrose (Pignocchi et al., 2021)] and less glucose or fructose (1 – 2.5 µg/mg FW). Therefore, it is very likely that pollen of Arabidopsis may use sugars as internal energy source for pollen germination and pollen tube growth if no apoplasmic sucrose is available.

We studied the impact of apoplasmic sucrose on pollen germination and pollen tube growth *in vitro* and observed very low germination rates on medium without sucrose and high germination rates on medium containing 250 mM sucrose. These data confirmed previous results that sucrose strongly promotes pollen germination (Li et al., 1999; Stadler et al., 1999; Palanivelu et al., 2003; Boavida and McCormick, 2007; Bou Daher et al., 2009; Rodriguez-Enriquez et al., 2013; Hirsche et al., 2017). *In vivo*, pollen germinates very well on the stigma. Stigmatic cells are symplasmically connected to sieve elements of the style which contains high amounts of sucrose [up to 0.3 M in the phloem (Deeken et al., 2002)]. Since sucrose exporting AtSWEET proteins are present in the stigma it can be assumed that pollen have access to high amounts of apoplasmic sucrose during germination on the stigma (Rottmann et al., 2018b).

However, if there is apoplasmic sucrose available in the transmitting tissue to feed the pollen during their growth towards the ovules is unknown. The *in vitro* growth assays showed that pollen tubes grew normally without any sucrose or in the presence of very high sucrose concentrations (150 – 300 mM). After 7 h *in vitro* growth, pollen tubes reached a very high length on medium containing 250 mM sucrose or 0 mM sucrose, whereas they stayed short on medium containing 50 mM sucrose. The data indicate that apoplasmic sucrose in concentrations higher than 50 mM and up to 300 mM enhances pollen tube growth. The presence of sucrose in lower concentrations (1 – 50 mM) led to a successive reduction in pollen tube length. This unexpected observation indicates that sucrose might not only serve as nutrient, but also as a signaling molecule for successful germination. Glucose for example can serve as a signaling molecule during pollen tube growth and even has an inhibitory effect, which disappears if fructose is present in an equimolar ratio (Rottmann et al., 2018b).

Interestingly, esculin assays with pollen tubes under low extracellular pH conditions demonstrated that sucrose carriers were active, however, pollen tubes did not grow and stayed short. Under higher pH conditions (pH 6.0 and above), sucrose carriers in pollen tubes were inactive but pollen tubes grew long. Generally, it is known for SUC carriers, which are H^+^ cotransporters, that they prefer low pH conditions (Sauer, 2007). The actual pH in the transmitting tissue is not known, and it is questionable if the growth inhibition at low pH values is physiologically relevant *in vivo*. However, the results indicate that the presence of external sucrose is more important for pollen germination than for the growth of pollen tubes.

Based on these data we developed the following hypothesis: Pollen need apoplasmic sucrose for efficient germination, while the uptake of sucrose is not necessary for pollen tube growth. Pollen may use apoplasmic sucrose for growth if it is available in concentrations above 50 mM. However, if there is no apoplasmic sucrose available, pollen may activate an alternative metabolic program to mobilize internal storages. This mobilization strategy may be inhibited by apoplasmic sucrose, however, the uptake of apoplasmic sucrose may be efficiently induced only if sucrose is present in concentrations of 150 to 300 mM. This would explain a vigorous growth under zero sucrose conditions and under very high sucrose concentrations, and a growth inhibition in the presence of medium sucrose concentrations. *In vivo*, pollen may face a situation without sucrose, for example when they land on top of each other on the stigma. However, if such a regulation actually occurs and has any physiological relevance *in vivo* remains speculative and needs further investigation.

### How pollen tubes fight for food – a model for the impact of sugar transport and sucrose cleavage on pollen performance

Generally, sucrose delivery from the staminal phloem to cells of the anther wall follows a symplasmic transport route *via* plasmodesmata (Imlau et al., 1999). Together with previous data, the results of the current work indicate that pollen performance depends on sugar availability, uptake, and cleavage (**Figure 7**). During gametogenesis, sugar uptake into pollen occurs in two steps (**Figure 7A**). The first step of sugar uptake takes place early during pollen development and includes the activity of a cytosolic invertase and the glucose exporter AtSWEET8/RUPTURED POLLEN GRAIN1 that passively releases monosaccharides from tapetum cells into the apoplasm in young flowers of stage 5 to 7 (Guan et al., 2008). The monosaccharides are taken up by AtSTP2, an active transport protein that accumulates glucose and fructose against a concentration gradient (Truernit et al., 1999). The second step occurs late during anther development in stage 12 flowers. In this stage, the tapetum is already degenerated. AtSWEET13 and AtSWEET14 unload sucrose from cells of the anther wall into the apoplasm (Wang et al., 2022). AtSUC1, which is also present in stage 12 flowers, catalyzes the uptake of sucrose through the plasma membrane of pollen. AtSUC1 activity causes very high sucrose concentrations in mature pollen, where the sucrose is most likely stored in the vacuole. Loss of AtSUC1 leads to a strongly reduced sucrose content in mature pollen and a defect in pollen germination (**Figure 7B**). Besides AtSUC1, pollen germination requires the invertase AtVI2, which hydrolyzes the vacuolar sucrose. The resulting monosaccharides are possibly exported into the cytosol and metabolized. Loss of AtVI2 causes a pollen germination defect. Growing pollen tubes express six genes for AtSUC sucrose transporters and six genes for AtSTP monosaccharide transporters (**Figure 7C**) (Rottmann et al., 2018a, b). Apoplasmic sucrose is either taken up directly by AtSUC proteins or after cleavage into monosaccharides by invertases AtcwINV2 and AtcwINV4 by AtSTPs (**Figure 7C** left). If no apoplasmic sucrose is available, pollen germination is severely reduced. However, the few pollen that manage to germinate grow normally, which indicates a metabolic reprogramming including the metabolization of internal energy reserves, possibly through the action of vacuolar invertases or sucrose transporters like AtSUC4, that transport sucrose from the vacuole into the cytoplasm (Schneider et al., 2012a).

## Data availability statement

The original contributions presented in the study are included in the article/**Supplementary Material**. Further inquiries can be directed to the corresponding author.

## Author contributions

RS conceived the research plans and supervised the experiments. JS and TR performed most of the experiments. CF generated the *AtSUC3*, *AtSUC5* and *AtSUC8* reporter constructs and performed the plant transformation, analyzed pollen germination and pollen tube growth tests, and performed the starch staining. CF, JK and TR performed the RT-qPCR analyses. CS generated the constructs for complementation lines. CS and WW performed genotyping of *Atvi2*. JS and RS designed the experiments and analyzed the data. JS wrote the article with the help of RS and with contributions of TR. RS, JS, TR, and CF discussed the data. All authors contributed to the article and approved the submitted version.
