# Supplementary material for: How pollen tubes fight for food: the impact of sucrose carriers and invertases of Arabidopsis thaliana on pollen development and pollen tube growth
Source: Front Plant Sci. 2023 Jun 22;14:1063765. doi: 10.3389/fpls.2023.1063765 (PMC10352115; doi:10.3389/fpls.2023.1063765)
Supplement: Supplementary file 1 [file DataSheet_1.pdf]

Supplementary material

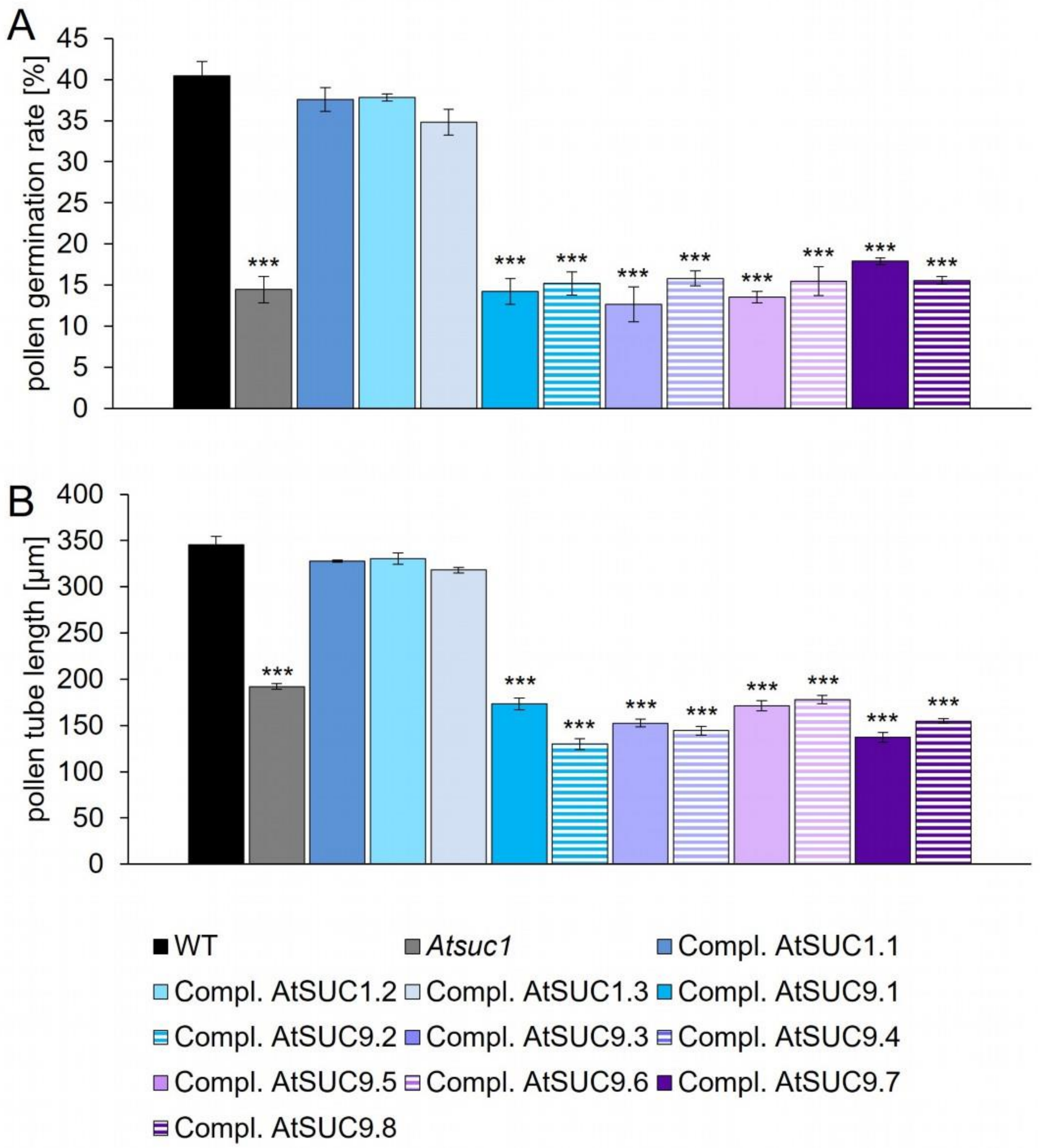

Supplementary Figure 1: Complementation of *Atsuc1* mutant line. (A) Pollen germination rates and **(B)** pollen tube lengths of complementation lines in the *Atsuc1* background compared to wild type pollen. Pollen germinated *in vitro* for 4 h on germination medium with 250 mM sucrose. For generating the complementation lines *Atsuc1* plants were respectively transformed by *Agrobacterium tumefaciens* with *AtSUC1*g under the control of the native *AtSUC1* promoter (Compl. AtSUC1.1 – 1.3) and *AtSUC9*g under the control of the native *AtSUC1* promoter (Compl. AtSUC9.1 – 9.8). Bars represent mean values (+/- SE) of three biological replicates (pollen germination rate: n > 500, pollen tube lengths: n > 200 for each genotype in each experiment). Significance: *Anova*. Reference: WT, **≙p ≤ 0.01; ***≙p ≤ 0.001.

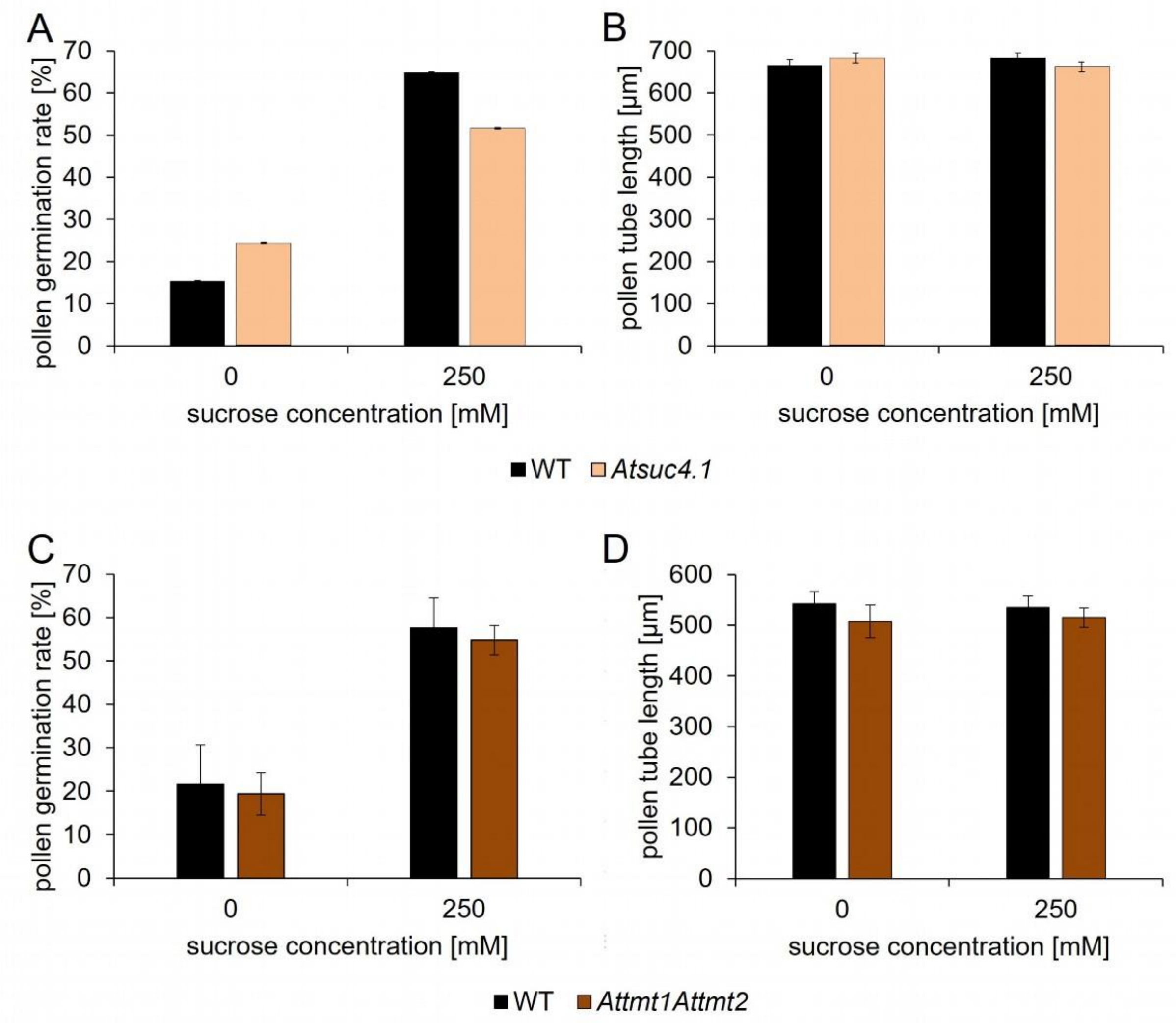

Supplementary Figure 2: Pollen germination and pollen tube growth tests with *Atsuc4.1* and *Attmt1Attmt2* pollen. **(A)** Pollen germination rates and **(B)** pollen tube lengths of *Atsuc4.1* and wild type pollen germinated *in vitro* for 7 h on pollen germination medium. Bars represent mean values (+/- SE) of three biological replicates (pollen germination rate: n > 500, pollen tube length: n > 200 for each genotype in each experiment). **(C)** Pollen germination rates and **(D)** pollen tube lengths of *Attmt1Attmt2* and WT pollen germinated *in vitro* for 7 h on pollen germination medium. Bars represent mean values (+/- SE) of three biological replicates (pollen germination rate: n > 500, pollen tube length: n > 200 for each genotype in each experiment). Significance: *Anova*. Reference: WT.

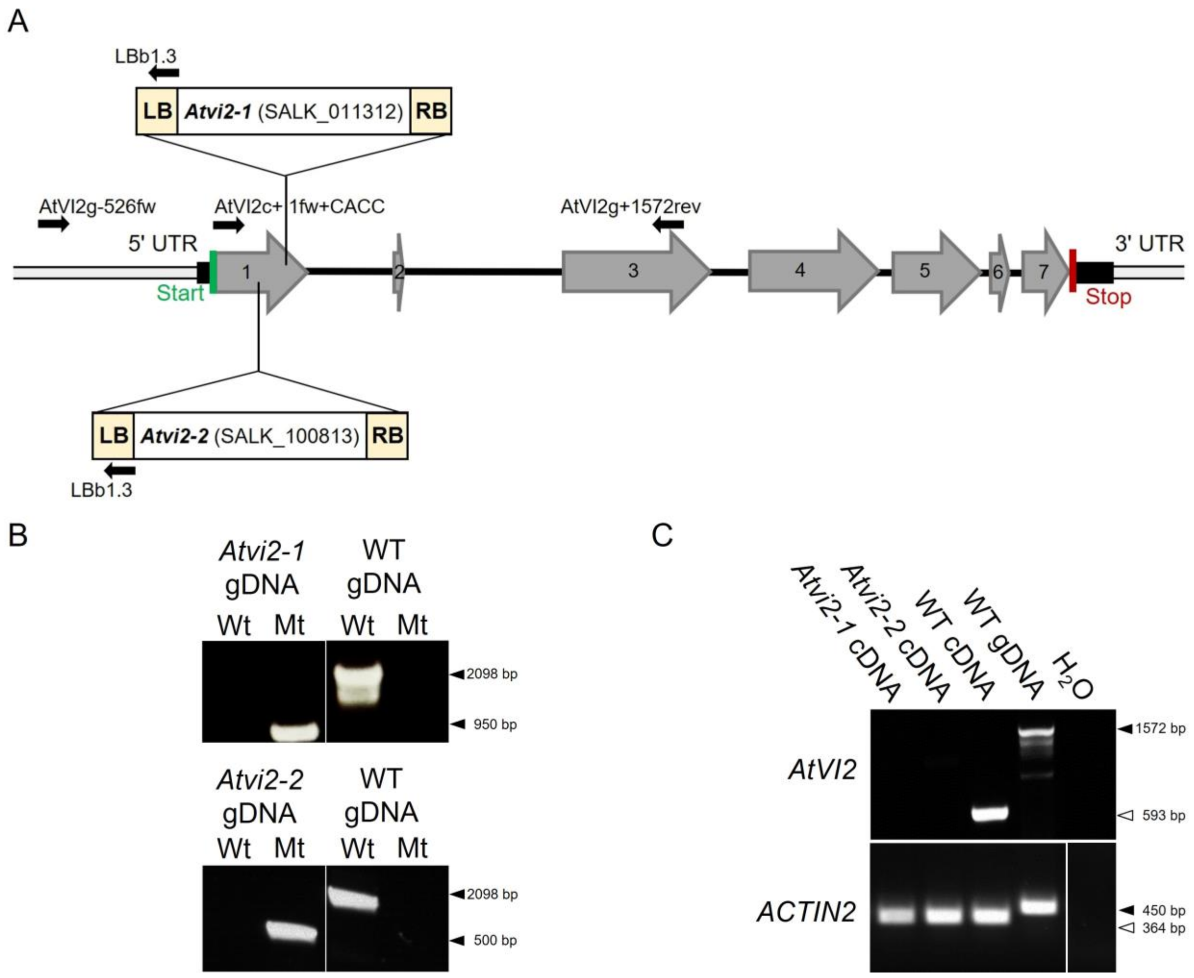

Supplementary Figure 3: Schematic overview and characterization of the *Atvi2-1* and *Atvi2-2* T-DNA insertions. **(A)** Schematic overview of the two T-DNA insertions *Atvi2-1* and *Atvi2-2*. Untranslated regions (UTR) and introns are shown as black lines, exons with coding regions as large gray arrows. The insertion of the T-DNA insertion line *Atvi2-1* starts +401 bp and the insertion of *Atvi2-2* +286 bp after start. Primers used for PCR reactions in B and C are shown with designation and orientation as small black arrows. **(B)** Genotyping of T-DNA insertion lines *Atvi2-1* and *Atvi2-2*. Genotyping was performed using genomic DNA isolated from leaves of *Atvi2-1*, *Atvi2-2*, and wild type. For the primer combinations of the respective *Atvi2-1* mutant bands (Mt), for the respective *Atvi2-2* mutant bands, and for the respective wild type (Wt) bands see Supplementary Table 5. Arrows indicate the size of PCR products derived from genomic DNA (black). **(C)** RT-PCR analyses using cDNA isolated from homozygous *Atvi2-1*, *Atvi2-2*, and wild type pollen, respectively. Genomic wild-type DNA was used as a control for contamination with genomic DNA, and amplification of the actin fragment (*ACTIN2*) served as an internal standard. For primer combinations see Supplementary Table 3. Arrows indicate the size of PCR products derived from reverse-transcribed mRNA (white) and genomic DNA (black).

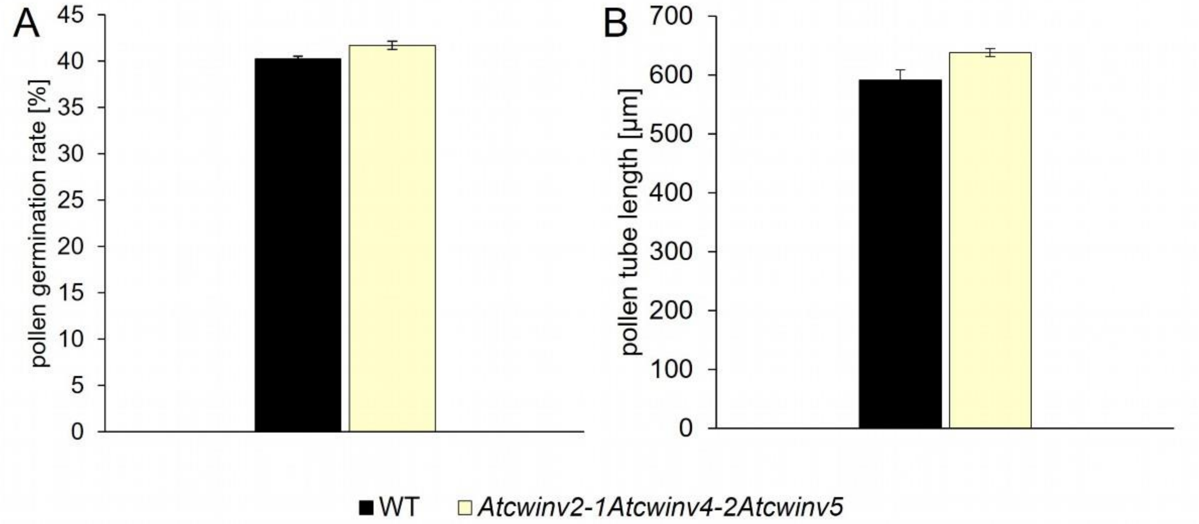

Supplementary Figure 4: Pollen germination and pollen tube growth tests with WT and *Atcwinv2-1Atcwinv4-2Atcwinv5* pollen. (A) Pollen germination rates and **(B)** tube lengths of wild-type and *Atcwinv2-1Atcwinv4-2Atcwinv5* pollen grains germinated *in vitro* for 7 h on pollen germination medium with 250 mM sucrose. Bars represent mean values (+/- SE) of three biological replicates (pollen tube length: n > 250, pollen germination rate n > 500 for each genotype in each experiment). Significance: *Anova*. Reference: WT.

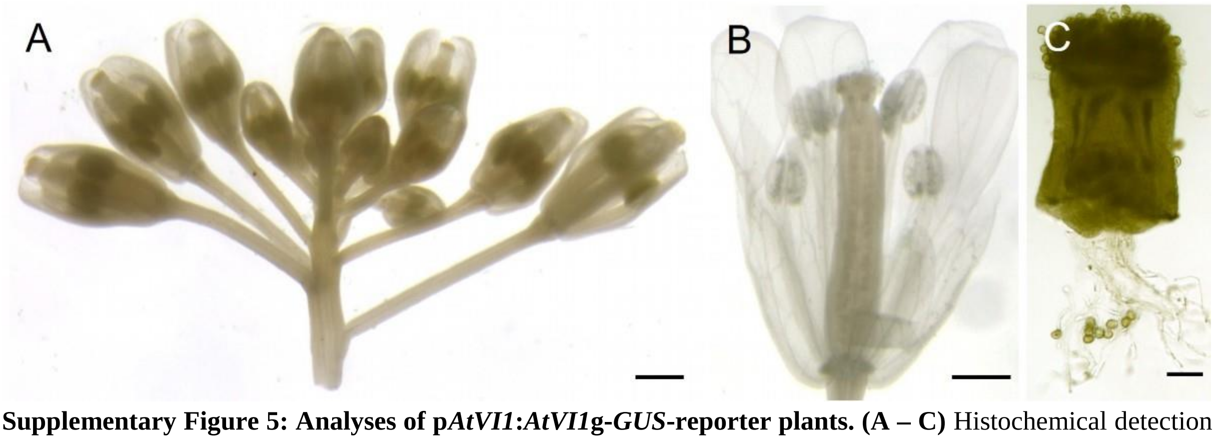

Supplementary Figure 5: Analyses of p*AtVI1*:*AtVI1*g-*GUS*-reporter plants. (A – C) Histochemical detection of ß-glucuronidase activity in *Arabidopsis thaliana* expressing *AtVI1*g-*GUS* under the control of the native *AtVI1* promoter. **(A)** Inflorescence. **(B)** Pollinated flower stage 14. **(C)** p*AtVI1*:*AtVI1*g-*GUS* pollen tubes germinated semi-*in vivo* on a WT stigma. Tissues were stained with GUS solution for 48 h **(A)** or 2 - 5 h **(B – C).** Scale bars: 500 µm in **(A, B)**; 125 µm in **(C)**.

Supplementary Table 1: Plant lines used in this study.

<table>
<tr><th>Name</th><th>Line</th><th>Gene</th><th>Reference</th></tr>
<tr><td>Arabidopsis thaliana Columbia-0 (Col-0)</td><td>-</td><td>-</td><td>Arabidopsis sequencing project (LEHLE SEEDS, Round Rock, USA)</td></tr>
<tr><td>Atcwinv2-1</td><td>SALK_068113</td><td>At3g52600</td><td>Alonso et al., 2003</td></tr>
<tr><td>Atcwinv4-2</td><td>SALK_130163</td><td>At2g36190</td><td>Alonso et al., 2003</td></tr>
<tr><td>Atcwinv5</td><td>SALK_GK849H10</td><td>At3g13784</td><td>Li et al., 2003</td></tr>
<tr><td>AtP5CR/Atp5cr-1</td><td>SALK_127043</td><td>At5g14800</td><td>Funck et al., 2012</td></tr>
<tr><td>Atsuc1</td><td>SM3_19978</td><td>At1g71880</td><td>John-Innes-Centre; Alonso et al., 2003</td></tr>
<tr><td>Atsuc3</td><td>GABIKat_325D02</td><td>At2g02860</td><td>Max-Planck-Institut; Alonso et al., 2003</td></tr>
<tr><td>Atsuc4.1</td><td>WiscDsLox450E10</td><td>At1g09960</td><td>Schneider et al., 2012b</td></tr>
<tr><td>Atsuc5.4</td><td>SALK_367_DO7</td><td>At1g71890</td><td>Pommerrenig et al., 2013</td></tr>
<tr><td>Atsuc8</td><td>SALK_066671</td><td>At2g14670</td><td>NASC, Loughborough, UK; Alonso et al., 2003</td></tr>
<tr><td>Atsuc9</td><td>SALK_050102</td><td>At5g06170</td><td>Sivitz et al., 2007</td></tr>
<tr><td>Attmt1</td><td>tmt1::tDNA</td><td>At1g20840</td><td rowspan="2">Wormit et al., 2006</td></tr>
<tr><td>Attmt2</td><td>tmt2::tDNA</td><td>At4g35300</td></tr>
<tr><td>Atvi2-1</td><td>SALK_011312</td><td rowspan="2">At1g12240</td><td rowspan="2">NASC, Loughborough, UK; Alonso et al., 2003</td></tr>
<tr><td>Atvi2-2</td><td>SALK_100813</td></tr>
</table>

Supplementary Table 2: Primers for PCR based genotyping of T-DNA insertion lines.

Mutant line	Amplified allele	Primer name	Primer sequence 5' → 3'
Atcwinv2-1	WT allele	Atcwinv2+1014fw	GACTTCAGCTACGACGGTCCAAA
		Atcwinv2+2193rev	CACTTTGCACCTTGGTTCATCTTCAG
	mutant allele	LBb1.3	ATTTTGCCGATTTCGGAAC
		Atcwinv2+2193rev	CACTTTGCACCTTGGTTCATCTTCAG
Atcwinv4-2	WT allele	Atcwinv4+1326fw	ACAATGAACGGTTCAGCATTC
		Atcwinv4+2515rev	ACATCCACAAAACCAGCAAAC
	mutant allele	Atcwinv4+1326fw	ACAATGAACGGTTCAGCATTC
		LBb1.3	ATTTTGCCGATTTCGGAAC
Atcwinv5	WT allele	AtcwINV5+36f	CTTGTTGGTCTCGTTGTTTCTCACTGAC
		AtcwINV5+1101rev	AACCGGCCATTGCACCAATCTC
	mutant allele	GabiKat LB	ATAATAACGCTGCGGACATCTACATTTT
		AtcwINV5+1101rev	AACCGGCCATTGCACCAATCTC
Atsuc1	WT allele	AtSUC1g+291fw	GGTCGCCGTCGTCCTTTCAT
		AtSUC1c+1544rev	AACTAGTGGAATCCTCCCATGGTCG
	mutant allele	Spm1	CTTATTTCAGTAAGAGTGTGGGGTTTGG
		AtSUC1c+1544rev	AACTAGTGGAATCCTCCCATGGTCG
Atsuc3	WT allele	AtSUC3g-129fw	TTCACCCGCCCGAATTTGACTC
		AtSUC3g+587rev	ATAGTTCCGAAAAAGGCTAAGGTC
	mutant allele	GabiKat LB	ATAATAACGCTGCGGACATCTACATTTT
		AtSUC3g+587rev	ATAGTTCCGAAAAAGGCTAAGGTC
Atsuc5	WT allele	AtSUC5g+978f	CGACCAAGGAGTTCAGGCTGG
		AtSUC5g+2061rev	TGACATGGCTGGTGCGTCCGG
	mutant allele	AtSUC5g+978f	CGACCAAGGAGTTCAGGCTGG
		LBb1.3	ATTTTGCCGATTTCGGAAC
Atsuc8	WT allele	AtSUC8-431fw	GTTTGTCGATTAACATCTGATTTC
		AtSUC8g+358rev	ATCCGATTAGTACAACAGCAACAG
	mutant allele	AtSUC8-431fw	GTTTGTCGATTAACATCTGATTTC
		LBa1	TGGTTCACGTAGTGGGCCATCG
Atsuc9	WT allele	AtSUC9-52fw	GATCGTGCGGCCGCCTTTTCATCTCCTCTATCACGATTCAC
		AtSUC9g+1258rev	GACATGGTGGACTTACAGCAAGAGG
	mutant allele	AtSUC9-52fw	GATCGTGCGGCCGCCTTTTCATCTCCTCTATCACGATTCAC
		LBa1	TGGTTCACGTAGTGGGCCATCG
Atvi2-1 & *Atvi2-2*	WT allele	AtVI2g-526fw	AATTCATATCATTGTCATACCCATTCAAACA
		AtVI2g+1572rev	GCTGAGCCGGTCCACACACC
	mutant allele	AtVI2g-526fw	AATTCATATCATTGTCATACCCATTCAAACA
		LBb1.3	ATTTTGCCGATTTCGGAAC

Supplementary Table 3: Primers for amplification of *AtSUC* transcripts in pollen tubes and stigmata by RT-PCR.

Gene	Primer name	Primer sequence 5’ → 3’
AtSUC1	AtSUC1c+983fw<br>AtSUC1c+1544rev	GTCTTGGAGTCCAATCTGGTGCAA<br>AACTAGTGGAATCCTCCCATGGTCG
AtSUC2	AtSUC2c+983fw<br>AtSUC2c+1573rev	GAGTCAGAGCTGGTGCTTTGGGG<br>GAGAGAAAGAGAGCCAAACAACCACTG
AtSUC3	AtSUC3c+1332fw<br>AtSUC3c+1783rev	TCGGGTTGTATGGGCTTTGAGC<br>AGCCGATGTGGAAACCGGTG
AtSUC4	AtSUC4c+998fw<br>AtSUC4c+1583rev	GTGCTGGGGTCAGTATGGGTGC<br>CTTATGCGTTCCTCGCTTGACACTC
AtSUC6	AtSUC6c+911fw<br>AtSUC6c+1521rev	GTCGTGAGGTGTACGGTGGAGAC<br>CATGTATGGGTCCTTATCTAGTGCTACAATAA
AtSUC7	AtSUC7c+1032fw<br>AtSUC7c+1507rev	TGGTATTGAAGGTATTAGTTAGGAAGATGGGA<br>GCATATATAATAAATAAAACGCATAAACTTTTTAAGG
AtSUC5	AtSUC5c+1193fw<br>AtSUC5c+1596rev	GACCGAGCAGTGGTATTAAAGCCG<br>TTTTGCACAACAATACTGTATTAGATGGAAATTC
AtSUC8	AtSUC8c+1033fw<br>AtSUC8c+1533rev	CTTGGTATTGAAGGTATTAGCAAGAAAATAGGT<br>GTATATAATTCACCTAGTACTACATTATATATTACAAGTAAAATG
AtSUC9	AtSUC9c+1029fw<br>AtSUC9c+1556rev	GTCCCTTGTTATTGGAGTGATTAGTAAGAAAAT<br>GCCACATGCTACATTAATATTAAACATGTGG
ACTIN2	AtAct2g+846f<br>AtAct2g+1295r	ATTCAGATGCCCAGAAGTCTTGTT<br>GAAACATTTTCTGTGAACGATTCCT

Supplementary Table 4: Primers for the amplification of *AtSUC*, *AtcwINV* and *AtVI2* transcripts over the respective T-DNA insertion by RT-PCR.

Mutant line	Primer name	Primer sequence 5’ → 3’
Atsuc1	AtSUC1+1fw+NotI<br>AtSUC1c+1544rev	GCGGCCGCATGGGAGCCTATGAAACAGAAAA<br>AACTAGTGGAATCCTCCCATGGTCG
Atsuc3	AtSUC3c+1fw+CACC<br>AtSUC3c+1244rev	CACCATGAGAAATTGATTTTGATACGAA<br>CGTACACCTTGATCATAGAGTTCCATATG
Atsuc8	AtSUC8+1fw+NotI<br>AtSUC8g+358rev	GCGGCCGCATGAGTGACCTCCAAGCAAAAAACG<br>ATCCGATTAGTACAACAGCAACAG
Atsuc9	AtSUC9g+348fw<br>AtSUC9g+1923rev	ACTAATTGGATTTGCGGCTGACTTC<br>AGGTAAAACGGTAAGTGCCACAACAC
Atcwinv2-1	AtcwINV2+1014fw<br>AtcwINV2+2193rev	GACTTCAGCTACGACGGTCCAAA<br>CACTTTGCACCTTGGTTCATCTTCAG
Atcwinv4-2	AtcwINV4+1360fw<br>AtcwINV4+2194rev	GGGCATTGGAGAACCGTGGTAG<br>GGTCCTACACCGCCTCTCACATTC
Atcwinv5	AtcwINV5+36fw<br>AtcwINV5+1101rev	CTTGTTGGTCTCGTTGTTTCTCACTGAC<br>AACCGGCCATTGCACCAATCTC
Atvi2-1 & *Atvi2-2*	AtVI2cds+1fw+CACC<br>AtVI2g+1572rev	CACCATGGCGAGCTCCGATGCTCTC<br>GCTGAGCCGGTCCACACACC

Supplementary Table 5: Primers for amplification of *AtSUC* transcripts in *Atsuc1*, Compl. AtSUC1, and Compl. AtSUC9 complementation lines and WT.

**Gene**	**Primer name**	**Primer sequence 5' → 3'**
p*AtSUC1*:*AtSUC1*g	AtSUC1-36fw<br>AtSUC1+1764rev	GTCTATTCGTGTTTTTTTTTTTTACTTCCTGA<br>GTGGAATCCTCCCATGGTCGTT
p*AtSUC9*:*AtSUC9*g	AtSUC9-58fw<br>AtSUC9+370rev	CCTCTATCACGATTCACCATCACCA<br>AGTCAGCCGCAAATCCAATTAGT
p*AtSUC1*:*AtSUC9*g	AtSUC1-36fw<br>AtSUC9+370rev	GTCTATTCGTGTTTTTTTTTTTTACTTCCTGA<br>AGTCAGCCGCAAATCCAATTAGT

Supplementary Table 6: Primers for qRT-PCR analysis of *AtSUC1*, *AtSUC3*, *AtSUC5*, *AtSUC6*, *AtSUC8* and *AtSUC9* transcript levels.

**Gene**	**Primer name**	**Primer sequence 5' → 3'**
AtSUC1	AtSUC1c+1315fw<br>AtSUC1c+1443rev	CAGGCCAAGGACTTTCATTAGGAG<br>TGCAACGATAAACGCCGGTAAG
AtSUC3	AtSUC3c+772fw<br>AtSUC3c+876rev	CAGCGTTTCTTCTTGCAGTGGTC<br>TTGTATGCGGGTGGGCTTGTTG
AtSUC5	AtSUC5c+1190fw<br>AtSUC5c+1258rev	CGGACCGAGCAGTGGTATTAAAGC<br>TGGCCAATGGGATACCAAGAACG
AtSUC6	AtSUC6c+1266fw<br>AtSUC6c+1326rev	GCGCTAGCTTCCATAATCTCTAGC<br>AACGCCTAGAGAGAGTCCTTGAC
AtSUC8	AtSUC8c+746fw<br>AtSUC8c+841rev	ATGGTCGCCTAAAGCAGACTCG<br>GCATCCACATTGGACGTTTCATC
AtSUC9	AtSUC9c+751fw<br>AtSUC9+818rev	CGCCTAATGCAGATTCCGACAATG<br>ACCTTGAAAGCTCCAAAGATCTCG
UBQ10	UBQ10+1066fw<br>UBQ10+1130rev	GATGGTCGTACTTTGGCGGATTAC<br>AGACGCAACACCAAGTGAAGGG

Supplementary Table 7: Cloning constructs for reporter gene lines used in this study.

Name	Insert	Destination vector	Promoter length [bp]	All exons and introns included	Reference
-	pAtSUC1:AtSUC1g-GUS	pBASTA-GUS	2000	✓	Sivitz *et al.*, 2008
pLEX1005	pAtSUC2:GUS	pBASTA-GUS	126	✓	Schneidereit *et al.*, 2008
pAL30	pAtcwINV5:AtcwINV5g	pBASTA-GUS	2168	✓	this study
pFC2	pAtSUC3:AtSUC3g	pBASTA-GUS	2174	✓	this study
pFC5	pAtSUC5:AtSUC5g	pBASTA-GUS	2118	✓	this study
pFC8	pAtSUC8:AtSUC8g	pBASTA-GUS	1544	✓	this study
pJS2	pAtcwINV1:AtcwINV1g	pBASTA-GUS	3028	✓	this study
pJS11	pAtVI2:AtVI2g	pBASTA-GUS	1296	✓	this study
pJS13	pAtcwINV2:AtcwINV2g	pBASTA-GUS	1107	✓	this study
pJS17	pAtcwINV4:AtcwINV4g	pBASTA-GUS	1415	✓	this study
pJS32	pAtSUC4:AtSUC4g	pBASTA-GUS	2044	✓	this study
pJS37	pAtVI1:AtVI1g	pBASTA-GUS	1933	✓	this study
pJS80	pAtSUC1:AtSUC9g	pMDC99	2025	✓	this study
pJS90	pAtSUC1:AtSUC1g	pMDC99	2025	✓	this study
pTR319	pAtSUC9:AtSUC9g	pBASTA-GUS	2159	✓	this study
pTR320	pAtSUC9:AtSUC9g	pBASTA-GFP	2159	✓	this study

Supplementary Table 8: Primers for generation of p*AtSUC*:*AtSUC*g-*GUS* and p*AtSUC*:At*SUC*g-*GFP* reporter gene lines.

Gene	Primer name	Primer sequence 5' → 3'
AtSUC3	AtSUC3-2174fw+CACC	CACCTTCAGGCGTAAGCATTTGACGTG
	AtSUC3g+1277rev	TTGGCAGGGGAGCAGGAGAG
	AtSUC3g+1199fw	CGGATTTTCTGCAGACATTGGG
	AtSUC3g+3748rev+A+AscI	TAGGCGCGCCTGCCGATGTGGAAACCGGTG
AtSUC4	AtSUC4-2044fw+CACC	CACCCTCCAGTTATGCCGCGAGCC
	AtSUC4g+2458rev	TGGGAGAGGGATGGGCTTCTG
AtSUC5	AtSUC5-2118fw+CACC	CACCCAACTAGAAAGTAATTCCCTCTACCCTCAG-3'
	AtSUC5g+93rev	CAGAGGAGATGGTTGACCGAGATC-3'
	AtSUC5g+43fw	GCCGCCAACAATGCTACGG
	AtSUC5g+2079rev+A+AscI	ATGGCGCGCCTATGGAATCCCATAGCCCCTGACA
AtSUC8	AtSUC8-1544fw+CACC	CACCCCTTAACCGAGGATCAATCCGG
	AtSUC8g+238rev	GACCAGAGACAGGACCACATAGCC
	AtSUC8g+191fw	CGTGCAGCTTCTTGGAGTCCC
	AtSUC8g+1709rev+A+AscI	TAGGCGCGCCTAGGTAACACGGTAAATGCCACAACA
AtSUC9	AtSUC9-2159fw+CACC	CACCCTGGTACATGGTTTGATTCACGTTTG
	AtSUC9g+1926rev	AGGTAAAACGGTAAGTGCCACAACAC

Supplementary Table 9: Primers for generation of p*AtcwINV*:*AtcwINV*g-*GUS* and p*AtVI*:*AtVI*g-*GUS* reporter gene lines.

Gene	Primer name	Primer sequence 5' → 3'
AtcwINV1	AtcwINV1-3028f_CACC	CACCACTCGGAGGATCGATGTTTTGCTAC
	AtcwINV1-1r_NcoI	CCATGGTGTGTGTATTTAATTTCTTTGTGGCTTGG
	AtcwINV1+1f_NcoI	ATCCATGGATGACCAAAGAAGTTTGCTCCAACATTG
	AtcwINV1+2745r_NcoI_ohne_Stop	ATCCATGGACTGATTTGGGCAGAGTTCATGCTC
AtcwINV2	AtcwINV2-1107fw+CACC	CACCTTCCGGTTTATCAACCAAATAGAAATGATT
	AtcwINV2+2191rev	CTTTGCACCTTGGTTCATCTTCAGAG
AtcwINV4	AtcwINV4-1415fw+CACC	CACCGAAATGGAATCAACATCACTAGCCAAA
	AtcwINV4+2832rev	AAGAGCTCCATCATTCATTTGCAGAG
AtcwINV5	AtcwINV5-2168fw+CACC	CACCCGAACGTGATAATAGTGGACTTTCAATG
	AtcwINV5-1rev+BamHI	GGATCCTGCAATCCTTTTTTTCGTGTTTATGTTTG
	AtcwINV5+1fw+BamHI	ATGGATCCATGGCTAATATAGTTTGGTGTAACATTGCC
	AtcwINV5+2138rev+ohneStop+BamHI	ATGGATCCAAGAGAAGACTTCATGCTCCAAGCAC
AtVI1	AtVI1g-1933 fw+CACC	CACCCCTGCCGGAATTGATTCTCGTTATAT
	AtVI1g+1698rev	GAGCATGACAATGGAGCCATCATCT
	AtVI1g+1474fw	CGTGTCCAGGTCCATTGTTCTATAAGG
	AtVI1g+3564rev+AscI+TA	ATGGCGCGCCGGTGCTGGAAGGAACACCGAGA
AtVI2	AtVI2-1296fw+CACC	CACCCATGTGCTTGACCAAACTAACAAGTGG
	AtVI2+3756rev	GGTACGGGAGAGAGCACGGACA

Supplementary Table 10: Primers for generation of the p*AtSUC*:*AtSUCg-pMDC99* complementation lines.

Gene	Primer name	Primer sequence 5' → 3'
p*AtSUC1*: *AtSUC1*g	AtSUC1-2025fw+CACC	CACCCGATTCCCAAAACACAGCTTAACC
	AtSUC1g+1764rev	AACGACCATGGGAGGATTCCAC
p*AtSUC1*: *AtSUC9*g	InFu1AtSUC1p(V)fw	AAGGGTGGGCGCGCCGACCCAGCTTTCTTGTACAAAGT
	InFu2AtSUC1p(V)rev	CATTATCAGGAAGTAAAAAAAAAAAACACGAATAGACAAAATAGAAACTTTAGA
	InFu3AtSUC9g(In)fw	TACTTCCTGATAATGAGTGACATCCAAGCAAAAGAAGATGCG
	InFu4AtSUC9g(In)rev	GGCGCGCCCACCCTTTTAAGGTAAAACGGTAAGTGCCAC
